# The role of 5-methylcytosine regulator-related genes in diagnostic and immune regulatory functions in atherosclerosis

**DOI:** 10.3389/fimmu.2025.1636323

**Published:** 2026-01-09

**Authors:** Hui Zhao, Ying Kong, Pengfei Ding, Luqun Yang, Ning Li

**Affiliations:** 1 Department of Geriatric Medicine, Shanxi Bethune Hospital, Shanxi Academy of Medical Sciences, Tongji Shanxi Hospital, Third Hospital of Shanxi Medical University, Taiyuan, China; 2 Department of Anesthesiology, Shanxi Bethune Hospital, Shanxi Academy of Medical Sciences, Tongji Shanxi Hospital, Third Hospital of Shanxi Medical University, Taiyuan, China; 3 Department of Cardiovascular Medicine, Shanxi Bethune Hospital, Shanxi Academy of Medical Sciences, Tongji Shanxi Hospital, Third Hospital of Shanxi Medical University, Taiyuan, China; 4 Graduate School of Shanxi Medical University, Taiyuan, Shanxi, China

**Keywords:** 5-methylcytosine, atherosclerosis, immune analysis, LASSO, macrophage

## Abstract

**Background:**

Atherosclerosis (AS) is a chronic inflammatory disease with a poor prognosis, and 5-methylcytosine (m5C) RNA modification plays a significant role in AS-induced cardio-cerebrovascular diseases (CCVDs). However, the effects of m5C modification and related genes in AS remain unclear.

**Methods:**

We analyzed the correlations between m5C RNA modification and its regulatory genes in AS using microarray databases. Specifically, microarray datasets of AS (GSE90074, GSE27034, GSE59421, and GSE159677) were obtained from the Gene Expression Omnibus (GEO) database. Following differential expression and Spearman correlation analyses using GSE90074, m5C regulator-related genes (MRRGs) were screened to construct a protein–protein interaction (PPI) network. A least absolute shrinkage and selection operator (LASSO) logistic regression model was constructed and validated using receiver operating characteristic (ROC) curves in GSE90074 and GSE27034 to identify feature genes. Consensus clustering analysis was then performed to classify AS samples into distinct clusters. In addition, Spearman correlation analysis was used to explore the associations between differentially expressed m5C regulators (DE-MRs) and immune cells based on the identified clusters. Weighted gene co-expression network analysis (WGCNA) was applied to identify AS-related module genes. Subsequently, intersecting genes common to module genes and differentially expressed genes (DEGs) across AS-related clusters were considered candidate biomarkers and were validated by quantitative real-time polymerase chain reaction (qRT-PCR) in human myeloid leukemia mononuclear cells (THP-1). Single-cell RNA sequencing (scRNA-seq) analysis was performed to characterize the immune microenvironment of AS. *In vitro* experiments and genetic interventions were conducted to investigate the effects of the m5C regulator NSUN3 on macrophage function. Finally, a competitive endogenous RNA (ceRNA) network targeting the identified biomarkers was predicted using the miRNet database.

**Results:**

Based on two differentially expressed m5C regulators (NSUN3 and NSUN5), 546 of 2247 DEGs between AS and control samples were identified as MRRGs for PPI network construction. Twenty hub MRRGs were further incorporated into the LASSO logistic regression model, yielding nine feature genes. Based on these feature genes, AS samples were classified into two clusters, with five immune cell types showing significant differences between clusters. Both NSUN3 and NSUN5 showed the strongest correlations with M0 macrophages. A total of 643 module genes were identified and overlapped with DEGs from the two AS-related clusters, resulting in five biomarkers—MCL1, F13A1, RGS2, Toll-like receptor 8 (TLR8), and TAGAP. The expression patterns of these five biomarkers in the foam cell model were consistent with those observed in public datasets. Furthermore, NSUN3 regulated the production of proinflammatory cytokines in macrophages. Finally, a ceRNA regulatory network was constructed.

**Conclusion:**

Five potential diagnostic biomarkers for AS—MCL1, F13A1, RGS2, TLR8, and TAGAP—were identified. In addition, the m5C regulator NSUN3 plays a critical role in macrophage function, providing experimental evidence that may support the diagnosis and treatment of AS.

## 1 Introduction

Cardiac-cerebral vascular diseases (CCVDs) are characterized by high morbidity and mortality and are responsible for nearly 20 million deaths annually worldwide (1). CCVDs, such as myocardial infarction and stroke, are primarily caused by atherosclerosis (AS), a complex chronic inflammatory vascular disease involving vascular endothelial dysfunction, inflammatory cell infiltration, oxidative stress, etc. Despite ongoing research, the precise pathogenesis of AS remains unclear. Thus, identifying reliable diagnostic biomarkers for AS and elucidating their associated modulatory networks are critical for early detection, assessment of disease severity, and ultimately, improved prognosis through stratified intervention.

Recent epigenetic studies have drawn attention to 5-methylcytosine (m5C) as a major focus in RNA methylation research. m5C is a ubiquitous modification in both coding and non-coding RNA. Messenger RNA (mRNA) sequencing in mice and human tissues has revealed a significant increase in m5C expression in heart and brain tissues compared with other organs. Gene ontology (GO) enrichment analysis has shown that m5C methylation is highly enriched in mRNA transcripts of mitochondrial-related genes (2). Due to high energy consumption and mitochondrial dependence, m5C methylation plays a crucial role in CCVDs by regulating mitochondrial functional balance.

The target RNA molecules modified by m5C methylation mainly include messenger RNA (mRNA), transfer RNA (tRNA), and ribosomal RNA (rRNA), resulting in diverse effects, such as influencing protein translation and participating in physiological processes associated with various diseases. Moreover, m5C modification sites are often embedded in CG-rich contexts and coding sequence (CDS) regions, thereby regulating diverse biological effects in AS. m5C modification is governed by m5C regulators, including “writers” (methyltransferases), “erasers” (demethylases), and “readers.” Among these, m5C writers mainly include NSUN1–7 and DNMT1–3; erasers include ALKBH1 and the TET family; and ALYREF and YBX1 serve as m5C readers that bind to m5C motifs. Despite some progress, the pathophysiological mechanisms underlying m5C regulators in CCVDs remain poorly understood. Intercellular adhesion molecule 1 (ICAM-1), a crucial factor in upregulating endothelial inflammation and vascular sclerosis, has been reported to be regulated by NSUN2-mediated m5C mRNA methylation (3). In addition, DNMT2, another critical m5C methyltransferase, participates in neuronal growth and development (4). Furthermore, Wang et al. demonstrated that parental zebrafish exposed to brominated diphenyl ethers (BDP) exhibited altered m5C DNA methylation patterns in key m6A regulatory components (methyltransferases, demethylases, and recognition proteins) within gonadal tissues. Notably, these epigenetic modifications were transgenerationally transmitted to offspring through germline inheritance, potentially disrupting vascular developmental processes. Mechanistically, BDP-induced methylation reprogramming impaired vascular morphogenesis by dysregulating epigenetic control of angiogenic signaling pathways, particularly through aberrant spatiotemporal expression of vascular endothelial growth factor (VEGF)-related genes (5, 6). However, the specific mechanisms of m5C regulators and related pathways in AS remain unclear.

In this study, we aimed to identify potential biomarkers for AS by analyzing m5C regulator-related genes (MRRGs). Based on feature genes obtained using a LASSO logistic model, AS samples were classified into distinct clusters. Through overlap analysis of AS-related module genes and differentially expressed genes (DEGs) across clusters, diagnostic biomarkers for AS were further identified. Moreover, the expression of these biomarkers was validated in human myeloid leukemia mononuclear cells (THP-1) using quantitative real-time polymerase chain reaction (qRT-PCR). The immune microenvironment characteristics of AS were explored using single-cell RNA sequencing (scRNA-seq) analysis. In addition, the role of the m5C regulator NSUN3 was investigated in an *in vitro* foam cell model. Finally, a competitive endogenous RNA (ceRNA) network of biomarkers was constructed using the miRNet database, which may provide insights into underlying mechanisms and serve as potential therapeutic targets for AS.

## 2 Materials and methods

### 2.1 Data collection

In this study, we utilized RNA-seq data related to AS obtained from the GEO cohort (https://www.ncbi.nlm.nih.gov/geo/) via the R package “GEOquery” (version 2.62.2). Specifically, the GSE90074 microarray, consisting of 93 AS and 50 healthy control blood samples, was used as the training set (**Table 1**), while the GSE27034 microarray, containing 19 peripheral artery disease (PAD) and 18 control peripheral blood mononuclear cell (PBMC) samples, was utilized as the validation set. The GSE159677 high-throughput sequencing dataset, containing three atherosclerotic core plaques and patient-matched adjacent portions of carotid artery tissue from patients undergoing carotid endarterectomy, was utilized as the single-cell sequencing dataset. In addition, we downloaded the GSE59421 microarray dataset from the Gene Expression Omnibus (GEO), which included microRNA (miRNA) expression data from 33 AS and 63 control blood samples. To identify m5C regulators and their associated genes, we referenced published articles and selected 17 m5C regulators, including 11 writers (NOP2, NSUN2, NSUN3, NSUN4, NSUN5, NSUN6, NSUN7, DNMT1, DNMT3A, DNMT3B, and TRDMT1), four erasers (TET1, TET2, TET3, and ALKBH1), and two readers (ALYREF and YBX1) (7–10).

**Table 1:** Clinical characteristics of the samples in GSE90074 (** p < 0.01, *** p < 0.001).

Characteristics	Control (n=50)	AS (n=93)	Total (n=143)	P-value
Gender, n(%)				0.004**
Female	32 (22.4%)	36 (25.2%)	68 (47.6%)	
Male	18 (12.6%)	57 (39.9%)	75 (52.4%)	
Ancestry, n(%)				0.543
AA	13 (9.1%)	20 (14.0%)	33 (23.1%)	
CAU	37 (25.9%)	73 (51.0%)	110 (76.9%)	
CAD_class, n(%)				<0.001***
0	18 (12.6%)	0 (0.0%)	18 (12.6%)	
1	32 (22.4%)	0 (0.0%)	32 (22.4%)	
2	0 (0.0%)	31 (21.7%)	31 (21.7%)	
3	0 (0.0%)	26 (18.2%)	26 (18.2%)	
4	0 (0.0%)	36 (25.2%)	36 (25.2%)	
Diabetes, n(%)				0.421
Yes	17 (11.9%)	38 (26.6%)	55 (38.5%)	
No	33 (23.1%)	55 (38.5%)	88 (61.5%)	
Hyperlipid, n(%)				0.003**
Yes	28 (19.6%)	74 (51.7%)	102 (71.3%)	
No	22 (15.4%)	19 (13.3%)	41 (28.7%)	
Hypertension, n(%)				0.976
Yes	44 (30.8%)	82 (57.3%)	126 (88.1%)	
No	6 (4.2%)	11 (7.7%)	17 (11.9%)	
CXCL5_rank, median(Q1, Q3)	72 (36,108)	98 (53,117.5)	61 (26.5,94)	0.002**
BMI, mean ± SD	30.00 ± 8.25	28.97 ± 6.27	29.35 ± 7.03	0.277

### 2.2 Analysis of differentially expressed m5C regulators and related genes

To identify differentially expressed genes (DEGs) between AS and control samples, we utilized the R package “limma” (version 3.50.1) with a p-value threshold of < 0.05 (11). We then visualized the expression of the top 10 upregulated and downregulated DEGs in a heatmap and compared the expression of m5C regulators between AS and healthy control samples to obtain DE-MRs.

Single-sample gene set enrichment analysis (ssGSEA) was used to calculate the score of each sample to evaluate the overall activity of the m5C-related regulatory network using the R package “GSVA” (version 1.42.0) (12). Correlations between DEGs and DE-MRs were calculated using Spearman correlation analysis with |Cor| > 0.3 and p < 0.05 to screen for m5C regulator-related genes (MRRGs). Moreover, the R package “clusterProfiler” (version 4.2.2) was used to enrich MRRGs into Gene Ontology (GO) functions and Kyoto Encyclopedia of Genes and Genomes (KEGG) pathways (13). Then, the STRING database (https://cn.string-db.org/) and the software “Cytoscape” (version 3.8.2) were used to generate a protein–protein interaction (PPI) network with a confidence score of 0.9 (14). The CytoHubba tool was applied to screen the top 20 hub MRRGs with the highest correlation degree from the network. Finally, the Comparative Toxicogenomics Database (CTD) (http://ctdbase.org/) was used to detect inference scores between the 20 hub MRRGs and AS incidence risk.

### 2.3 Construction of a LASSO logistic model

The R package “glmnet” (version 4.1-2) was applied to construct a logistic regression model using MRRGs to identify genes significantly associated with AS (p < 0.001 and OR≠1) (15). These genes were defined as feature genes, followed by further filtering using the minimum value of lambda (λ) in the LASSO algorithm. Next, the R package “pROC” (version 1.18.0) was used to plot receiver operating characteristic (ROC) curves based on both the training and validation sets to evaluate the prognostic performance of the model (16). Moreover, to identify miRNAs and transcription factors (TFs) regulating feature genes, a TF–mRNA–miRNA network was constructed using the miRNet database (https://www.mirnet.ca/) based on gene expression levels in the model.

### 2.4 Consensus clustering analysis and co-expression analysis

Consensus clustering analysis was performed using the R package “ConsensusClusterPlus” (version 1.58.0) based on the expression of nine feature genes and the optimal k value to classify AS samples in the training set into distinct clusters (17). To verify the separability of sample distributions between clusters, principal component analysis (PCA) and t-distributed stochastic neighbor embedding (t-SNE) analysis were conducted using the R packages “factoextra” (version 1.0.7), “FactoMineR” (version 2.4.0), and “Rtsne” (version 0.15).

Next, the R package “Weighted Gene Co-expression Network Analysis (WGCNA)” (version 1.70-3) was employed to identify modules with the highest correlation with different clusters using the training set. A hierarchical clustering tree was generated to remove outlier samples, and the most suitable soft-threshold power (β) was selected to construct an adjacency matrix. The adjacency matrix was then transformed into a topological overlap matrix (TOM), and genes with similar expression patterns were clustered into the same module. Furthermore, relationships between co-expression modules and clusters were calculated using Pearson’s correlation coefficient and visualized using a heatmap.

### 2.5 Screening and immune analysis of biomarkers of AS

Differentially expressed genes between clusters were obtained using the R package “limma” based on p < 0.05 and |log_2_FC| > 0.5 (18). Enrichment analysis was performed to identify GO functions and KEGG pathways associated with these DEGs. Moreover, immune cell composition in AS samples was inferred using the R package “CIBERSORT,” and correlations between immune cell levels and DE-MRs were examined (19).

Candidate biomarkers were identified by intersecting DEGs with genes from WGCNA-selected modules. Expression levels of candidate biomarkers were compared between AS and control samples in both the training and validation sets, and correlation analysis was conducted between feature genes in the LASSO logistic model and biomarkers. In addition, correlations between biomarkers and drugs were analyzed using the Drug–Gene Interaction Database (DGIdb) (http://www.dgidb.org/). Finally, a TF–mRNA–miRNA network was generated using the miRNet database.

### 2.6 Cell cultures and foam cell model construction

Human myeloid leukemia mononuclear cells (THP-1) were obtained from America Type Culture Collection (ATCC, USA). Cells were cultured in RPMI 1640 medium containing 10% FBS and 1% penicillin-streptomycin (Beyotime, Shanghai, China) in a humidified atmosphere of 5% CO_2_ at 37°C. THP-1 cells were seeded into 6-well plates at a density of 1×10^6^cells per well and designated as the control group. To construct the foam cell model, THP-1 cells were exposed to phorbol myristate acetate (100nM, Sigma-Aldrich, Merck KGaA) for 48h, and then incubated with 100μg/ml ox-LDL for 24h.

### 2.7 qRT-PCR

Total RNA was extracted from cultured cells using the High Purity Total RNA Extraction Kit (BioTeKe, Beijing, China) according to the manufacturer’s instructions. RNA concentration and purity were measured using a NanoDrop 3000 spectrophotometer (Thermo Fisher Scientific, Scotts Valley, CA, USA). Complementary DNA was synthesized using the High-Capacity cDNA Reverse Transcription Kit (Thermo Fisher Scientific, Waltham, MA, USA). Primers used in this study were purchased from Sangon Biotech Co., Ltd. (Beijing, China). Transcripts were quantified using SYBR Green (Roche, Mannheim, Germany) on an ABI 7900 Fast Real-Time PCR System (Applied Biosystems, Foster City, CA, USA) for 45 cycles. Relative mRNA expression was analyzed using the 2-△△Ct method. The following primer sequences were used (F: forward; R: reverse):

MCL1 F: 5’-GCTTCGGAAACTGGACATCA-3.

MCL1 R: 5’-GGAAGAACTCCACAAACCCATC-3.

F13A1 F: 5’-GTCACGAGCGTTCACCTGTT-3.

F13A1 R: 5’-TTGTTCTCCTGTGGGTAGCG-3.

RGS2 F: 5’-ACCACAGAGCCTCATGCTAC-3.

RGS2 R: 5’-GACACCACGTTCAGACCACC-3.

TLR8 F: 5’-TGACTTTACATCTTCCCTTCGG-3.

TLR8 R: 5’-AAGTGCGGATTTGTTGATTGTT-3.

TAGAP F: 5’-TGTCATTTGAAGCCCAGAAGG-3.;

TAGAP R: 5’-ATCAGGGTCGTTGCTGTCGTA-3.

### 2.8 scRNA-seq analysis

The GSE159677 dataset was processed using the R package “Seurat” (version 5.1.0). Quality control was performed by retaining cells with the number of detected genes (nFeature) between 200 and 6000, the total number of RNA molecules (nCount) between 500 and 20000, and the percentage of mitochondrial genes (percent.mt) below 10%. Logarithmic normalization was subsequently applied, and 2000 highly variable genes were selected using the FindVariableFeatures function for downstream analysis. Principal component analysis (PCA) was performed using the ElbowPlot function to determine the optimal number of principal components. The FindNeighbors function was applied to construct a cell neighborhood graph, and the FindClusters function (resolution = 0.4) was used to identify cell subpopulations for fine-grained clustering analysis. Based on the clustering results, the uniform manifold approximation and projection (UMAP) algorithm was employed for dimensionality reduction and visualization. The FindAllMarkers function was then used to identify cluster-specific highly expressed genes (min.pct = 0.25, logfc.threshold = 0.1). Cell clusters were annotated into different cell types using the R package “SingleR” (version 2.2.0) combined with the CellMarker database, and the annotated cell types were visualized using UMAP plots. Furthermore, the expression patterns of five biomarkers and two DE-MRs (NSUN3 and NSUN5) across the identified cell types were visualized using the R package “ggplot2” (version 3.3.5).

### 2.9 Virus package and cell transfection

293T cells (3.8×10^6^ per 100 mm dish) were seeded into culture plates. After culturing for 24 h, a mixture of plasmids, including the target plasmids (PCDH plasmid and PCDH-NSUN3 plasmid) and additional packaging plasmids (Addgene, Cambridge, MA, USA), was transfected into the cells using Lipofectamine 3000 (Invitrogen, Carlsbad, CA, USA). Viruses were harvested and concentrated at 72 h post-transfection. THP-1 cells were seeded into 6-well plates at a density of 2×10^5^ cells per well. The virus containing NSUN3 was used to infect THP-1 cells to induce NSUN3 overexpression, while a control virus served as the negative control. After 24 h of incubation, the viral medium was replaced with fresh medium, and THP-1 cells were cultured for an additional 48 h. Phorbol 12-myristate 13-acetate (PMA) was added to induce differentiation of THP-1 cells into macrophages (M0) for 48 h, followed by ox-LDL treatment to stimulate foam cell formation.

### 2.10 Flow cytometry

Anti-human NSUN3 antibody (Thermo Fisher Scientific, Lenexa, KS, USA), anti-human NSUN5 antibody (Thermo Fisher Scientific, Lenexa, KS, USA), Annexin V-FITC/PI Staining Detection Kit (BD Pharmingen, San Diego, CA, USA), anti-human CD86 antibody (BioLegend, Beijing, China), and anti-human CD206 antibody (BioLegend, Beijing, China) were used according to the manufacturer’s instructions. After harvesting and washing cells with PBS three times, cells were digested with EDTA-free trypsin. Cells were stained with NSUN3 antibody, NSUN5 antibody, Annexin V-FITC, propidium iodide (PI), CD86 antibody, and CD206 antibody for 25 min in the dark. FlowJo software was used for data acquisition and analysis.

### 2.11 Cytokine array

Cells were collected and lysed using the Human XL Cytokine Array Kit (R&D Systems, Minneapolis, MN, USA). Protein extracts were adjusted to a concentration of 1 mg/mL for each test according to the manufacturer’s instructions. After incubation with antibodies and streptavidin–HRP conjugate, dot images were captured using a Bio-Rad ChemiDoc XRS system. Spot intensities were assessed using ImageJ software and normalized to the average values of control spots.

### 2.12 Construction of a ceRNA network

Differentially expressed microRNAs (DE-miRNAs) between AS and control samples were identified using the R package “limma,” with p < 0.05 as the significance threshold (11). Long noncoding RNAs (lncRNAs) with reciprocal relationships were screened by intersecting miRNAs obtained from the miRNet database with DE-miRNAs. Finally, a ceRNA network was constructed based on miRNA and lncRNA interaction information.

### 2.13 Statistical analysis

Statistical analyses were performed using R software (version 4.1.0). The R packages “ggplot2” (version 3.3.5) and “pheatmap” (version 1.0.12) were used to generate volcano plots, box plots, violin plots, and heatmaps. Forest plots were generated using the R package “forestplot” (version 2.0.1), and ROC curves were plotted using the R package “geomROC” (version 1.0). scRNA-seq data were analyzed using the R packages “Seurat” (version 5.1.0) and “SingleR” (version 2.2.0). Data are presented as mean ± standard deviation (SD), and cellular experiments were repeated 3–5 times. Differences between two groups were analyzed using a two-tailed Student’s t-test. Nonparametric tests were applied for non-normally distributed variables. Spearman correlation analyses between candidate gene expression levels and key clinical continuous parameters were conducted using IBM SPSS Statistics (version 26.0). Statistical analyses were also performed using GraphPad Prism 10.0 (CA, USA). A p-value < 0.05 was considered statistically significant.

## 3 Results

### 3.1 –2247 DEGs and two DE-MRs between AS and healthy control samples

**Figure 1:**
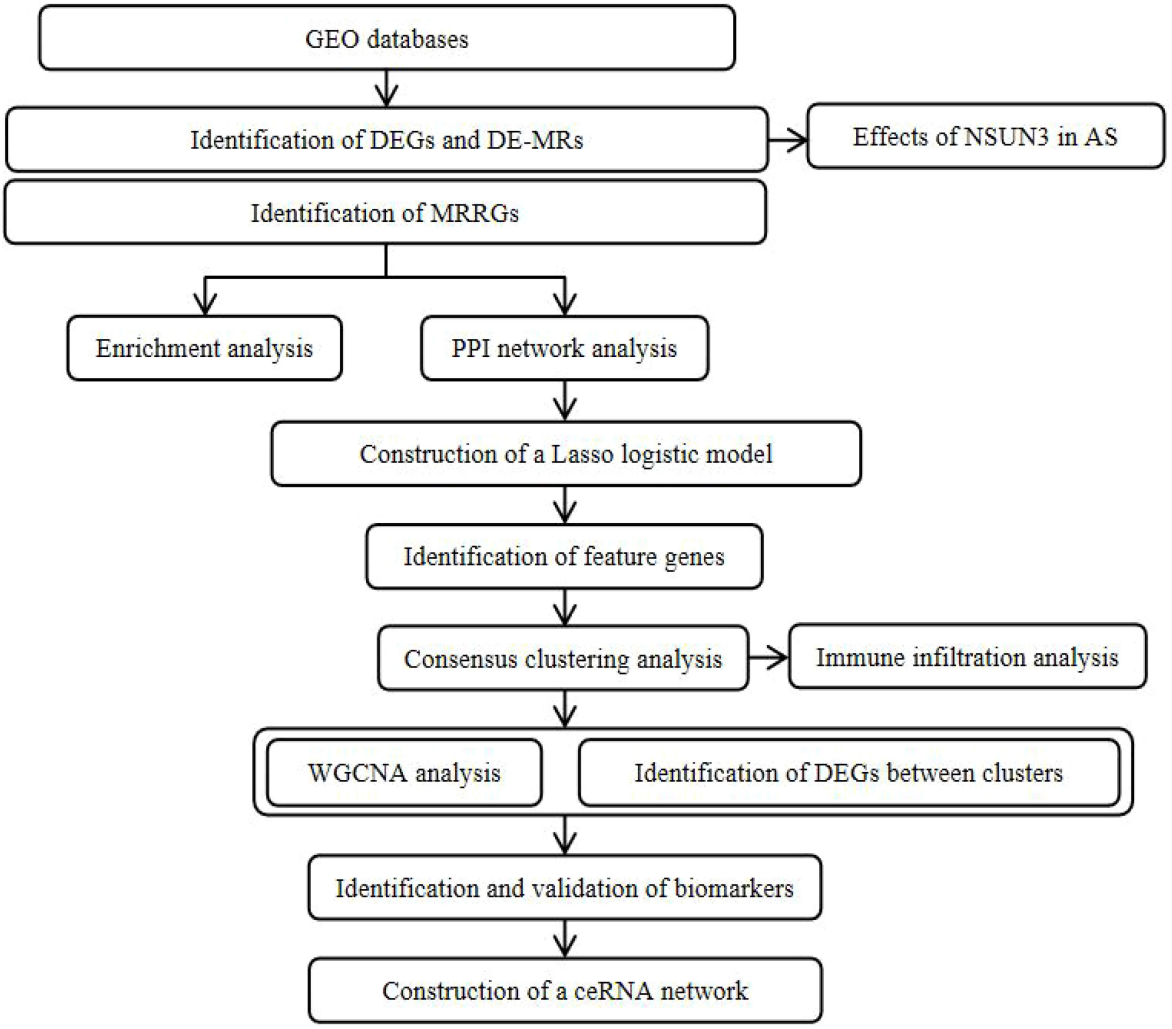
Graphical abstract of the current study.

**Figure 2:**
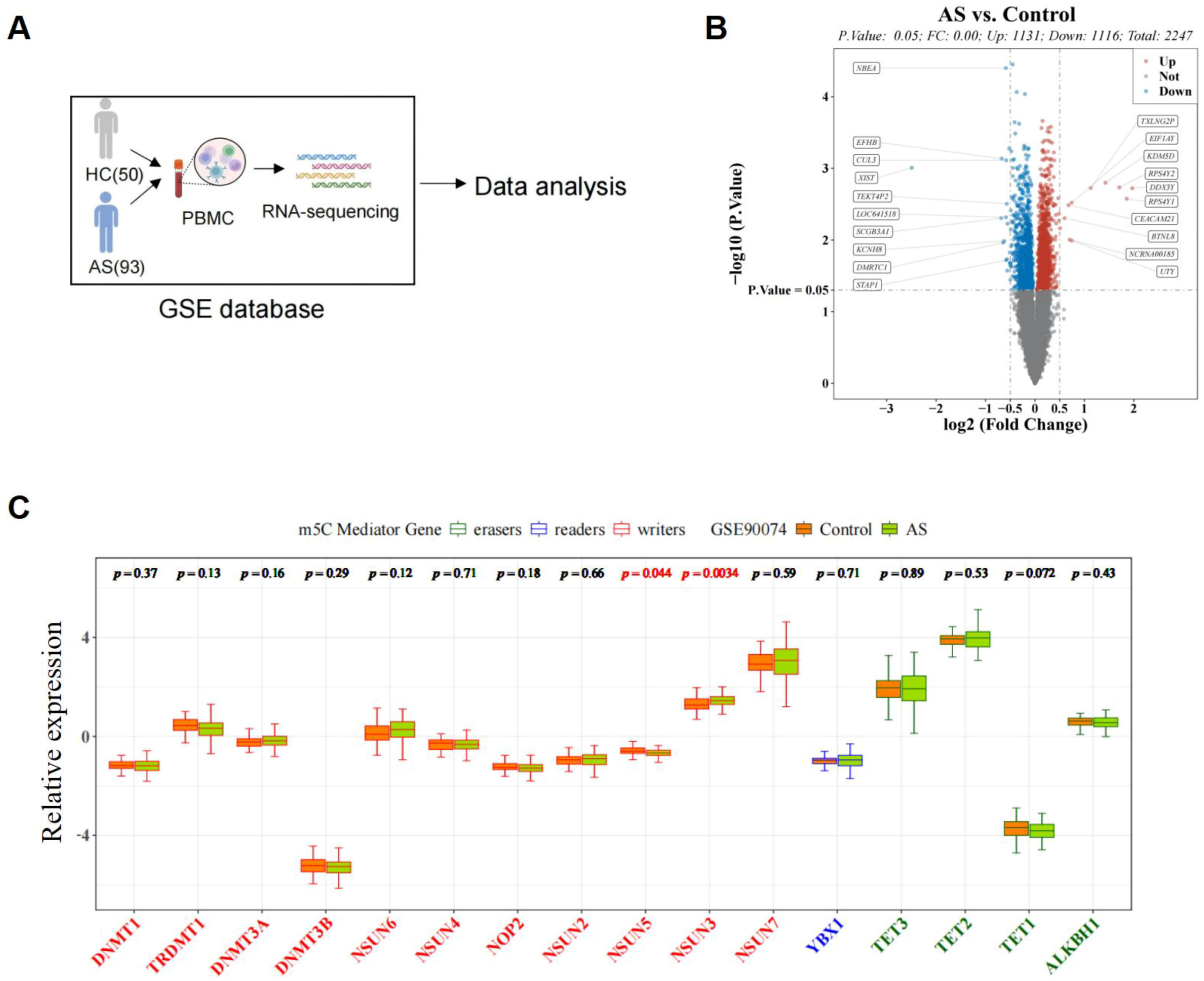
Identification of DEGs and DE-MRs between AS and healthy control samples in the datasets. **(A)** Flowchart of bioinformatics analyses in GSE90074 database. **(B)** Volcano plot showed DEGs between AS and control samples. **(C)** Expression of DE-MRs between AS and control samples.

The graphical abstract is shown in **Figure 1**. We performed differential expression analysis on microarray data from GSE90074 (AS = 93 and control = 50 blood mononuclear samples) and identified 2247 DEGs, including 1131 upregulated and 1116 downregulated genes (**Figure 2A**). The volcano plot in **Figure 2B** and **Supplementary Table S1** display the top 10 DEGs ranked by logFC, including TXLNG2P, KDM5D, RPS4Y2, DDX3V, RPS4Y1, EIF1AY, CEACAM21, BTNL8, NCRNA00185, and UTY as upregulated genes, and NBEA, EFHB, CUL3, XIST, TEKT4P2, LOC641518, SCGB3A1, KCNH8, DMRTC1, and STAP1 as downregulated genes. We also compared the expression of 17 m5C regulators between AS and control samples and identified two m5C regulators, NSUN3 and NSUN5, that showed significant differences (**Figure 2C**).

### 3.2 –546 MRRGs were obtained via correlation analysis

**Figure 3:**
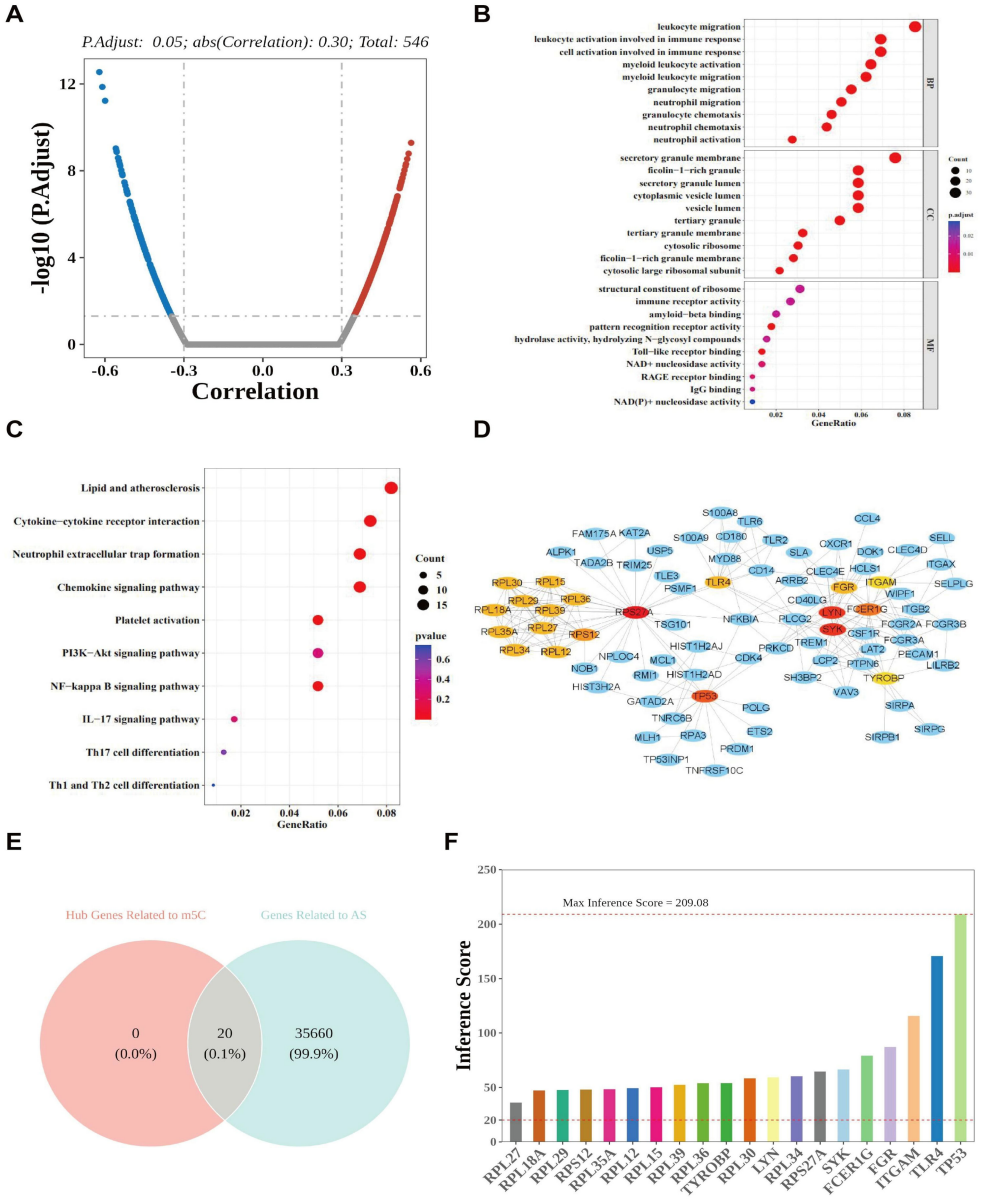
dentification of MRRGs via correlation analysis. **(A)** DEGs between AS and control groups showing strong correlation with DE-MRs (|Cor| > 0.3). GO enrichment**(B)** analysis of MRRGs. **(C)** KEGG pathway analysis of MRRGs. **(D)** PPI network based on 20 hub MRRGs and their correlated genes. **(E)** Venn diagram of 20 AS-related hub genes. **(F)** Bar plot showing inference scores between the 20 hub MRRGs and AS incidence risk.

To evaluate the overall activity of the m5C-related regulatory network, we used the ssGSEA method to calculate the score of each sample based on GSE90074 (**Supplementary Table S2**). This method calculated enrichment scores for potential target gene sets regulated by NSUN3 and NSUN5 within each sample, providing a quantitative measure of their regulatory activity. Spearman correlation analysis was then performed between DEGs and DE-MRs, and 546 DEGs showing strong correlations (|Cor| > 0.3) with DE-MRs were identified (**Figure 3A**). These genes were defined as m5C regulator-related genes (MRRGs). The MRRGs were enriched in 305 Gene Ontology biological process (GO-BP) terms, including “leukocyte migration” and “leukocyte activation in immune response”; 30 GO cellular component (GO-CC) terms, such as “secretory granule membrane” and “ficolin-1-rich granule”; and 15 GO molecular function (GO-MF) terms, including “structural constituent of ribosome” and “immune receptor activity” (**Figure 3B**). In addition, these MRRGs were significantly enriched in 241 Kyoto Encyclopedia of Genes and Genomes (KEGG) pathways, including “lipid and atherosclerosis,” “cytokine–cytokine receptor interaction,” “neutrophil extracellular trap formation,” and “chemokine signaling pathway” (**Figure 3C**). A comprehensive summary of the enrichment analysis is provided in **Supplementary Tables S3** and **Supplementary Tables S4**.

To explore the molecular mechanisms underlying AS incidence, we constructed a protein–protein interaction (PPI) network based on the 546 MRRGs (**Supplementary Figure S1**). From this network, the top 20 MRRGs ranked by connectivity degree (k) were identified (**Supplementary Figure S2**; **Supplementary Table S5**) and defined as hub MRRGs. These included RPS27A, LYN, SYK, TP53, FCER1G, RPS12, RPL18A, RPL15, RPL39, RPL36, RPL27, RPL35A, RPL30, RPL34, RPL29, FGR, Toll-like receptor 4 (TLR4), RPL12, ITGAM, and TYROBP. Notably, three hub MRRGs—RPS27A, LYN, and SYK—were each correlated with more than 18 genes (**Figures 3D**; **Supplementary Figure S3**). Although ribosomal proteins frequently appear as hubs in PPI analyses due to their high and coordinated expression, the network also identified several non-ribosomal hub genes with established roles in immune and inflammatory processes, including LYN, SYK, and TLR4, which are directly relevant to AS.

To further assess the relevance of these hub MRRGs to AS, inference scores between the 20 hub MRRGs and AS incidence were calculated using the Comparative Toxicogenomics Database (CTD). TP53 and TLR4 exhibited relatively high inference scores, both exceeding 150 (**Figures 3E, F**).

Overall, PPI network analysis of m5C-related genes confirmed multiple genes with established roles in immune and inflammatory processes in AS, such as LYN, SYK, and TLR4, and further demonstrated strong associations between core genes, including TP53 and TLR4, and AS based on CTD analysis. From a systems biology perspective, these results indicate that the m5C-related gene network is deeply involved in immune and inflammatory mechanisms underlying AS, providing a foundation for subsequent immune analyses and enabling the identification of hub genes closely associated with both m5C regulation and AS.

### 3.3 Nine feature genes in the LASSO logistic model of AS

**Figure 4:**
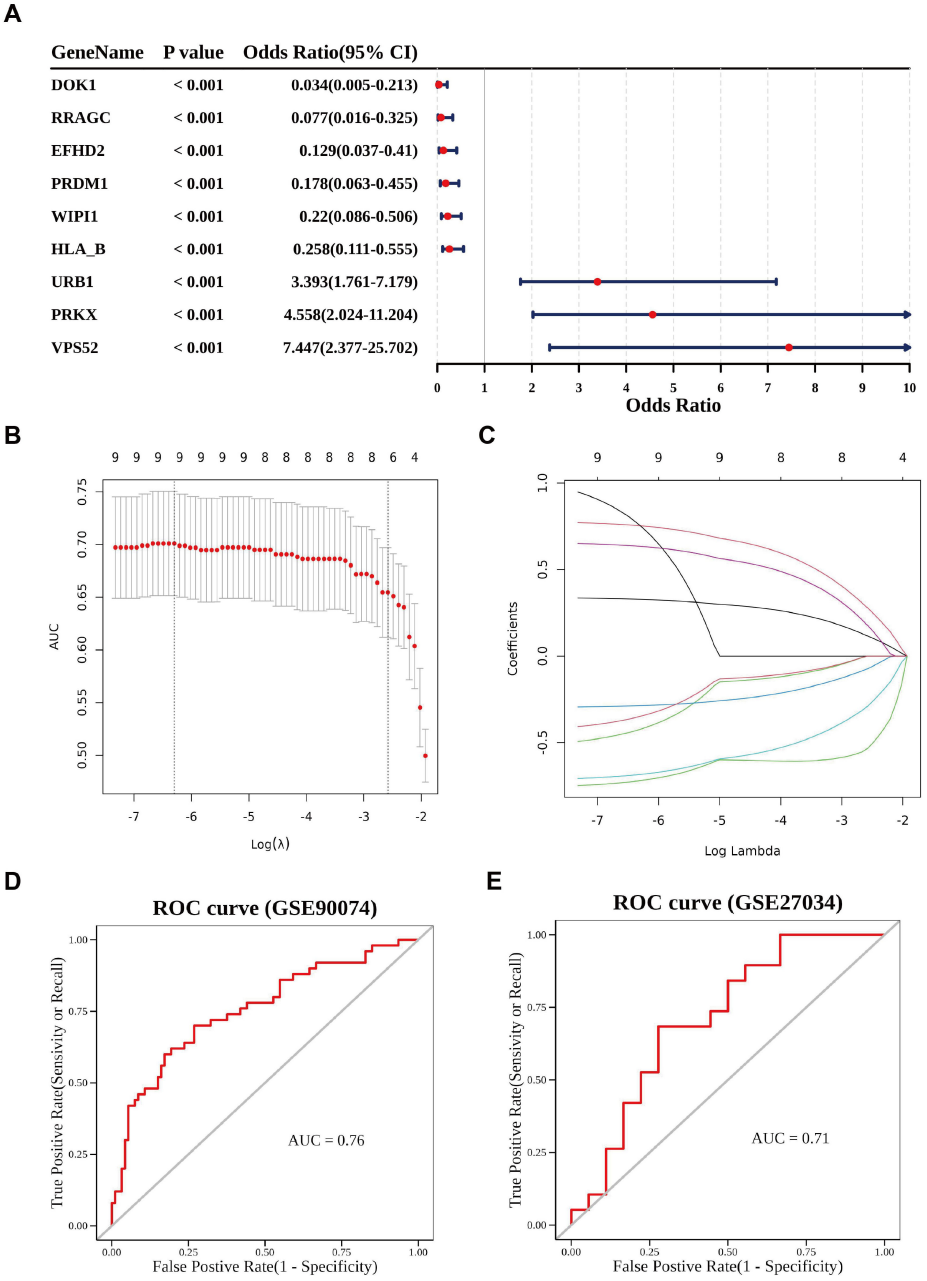
Feature genes in the logistic regression model of AS. **(A)** Feature genes selected by the univariate Cox regression analysis. **(B)** Cross-validation for tuning parameter selection in the LASSO model. **(C)** Feature genes selected by the LASSO model. **(D)** Predictive performance of the model in GSE90074 (ROC curves). **(E)** Predictive performance of the model in GSE27034 (ROC curves).

To construct a diagnostic model for AS, feature selection was performed on the 546 MRRGs using a LASSO logistic regression model. This analysis identified nine feature genes: DOK1, RRAGC, EFHD2, PRDM1, WIPI1, HLA-B, URB1, PRKX, and VPS52 (**Figures 4A–C**). To evaluate the diagnostic performance of the model, receiver operating characteristic (ROC) curves were generated for both the training set (GSE90074) and the validation set (GSE27034). The area under the curve (AUC) values for both datasets exceeded 0.7, indicating that the model has a reasonable ability to distinguish peripheral arterial disease (PAD) samples from control samples (**Figures 4D, E**).

### 3.4 Cluster analysis of AS samples and identification of gene modules with WGCNA

**Figure 5:**
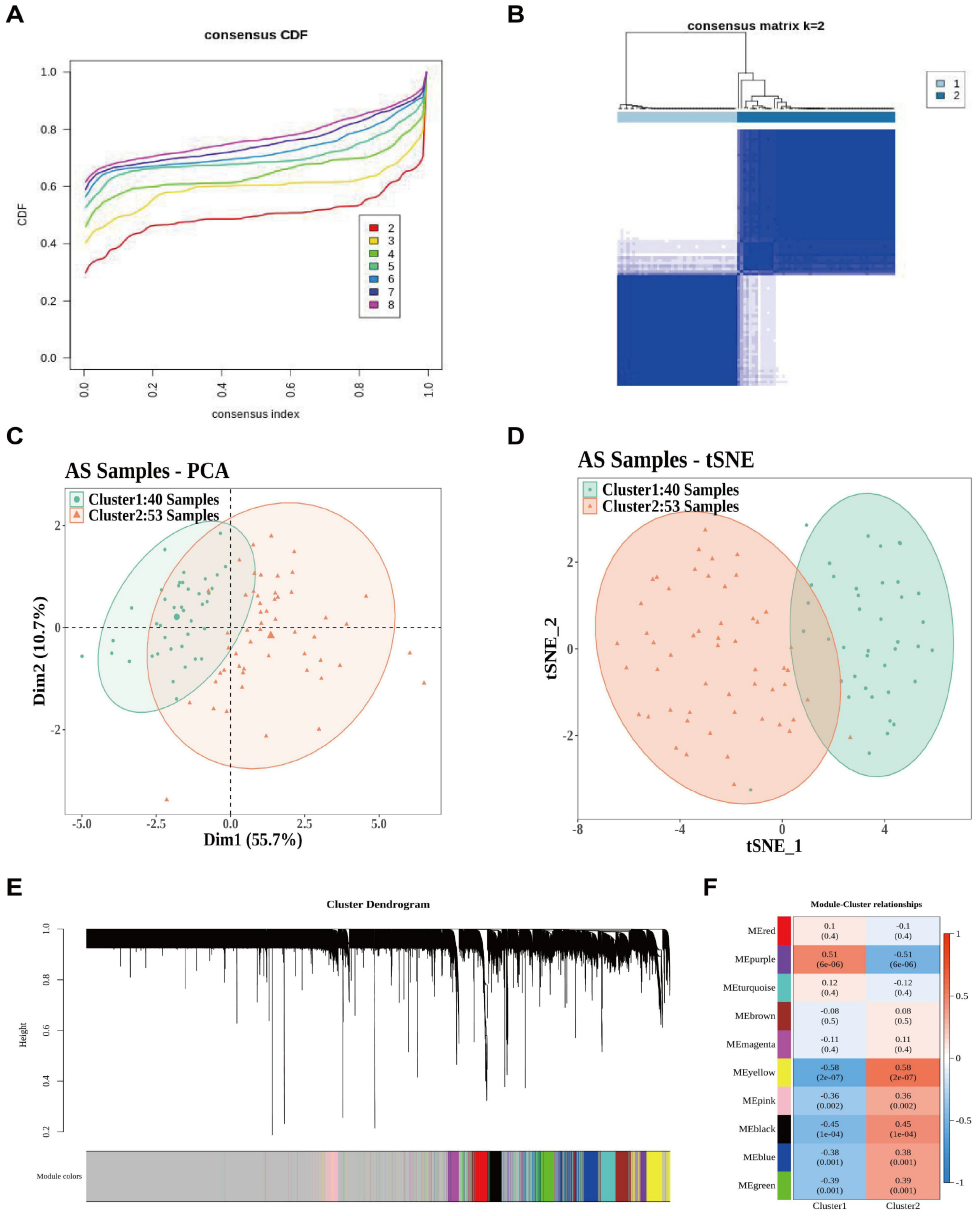
Consensus clustering analysis. **(A)** Consensus clustering cumulative distribution function **(CDF)** when data were divided into 2–9 clusters. **(B)** AS samples divided into two clusters based on the expression of nine feature genes (k = 2). **(C)** PCA validation of cluster separation. **(D)** tSNE validation of cluster separation. **(E)** Module clustering dendrogram derived from the TOM. **(F)** Module–cluster relationships in AS.

We utilized consensus clustering analysis to classify AS samples into distinct subclusters based on the expression of nine feature genes. Using the consensus clustering heatmap and cumulative distribution function (CDF) curve, we determined an optimal k value of 2, which separated the samples into cluster 1 and cluster 2 (**Figures 5A, B**). Further, the distribution of samples in cluster 1 was significantly different from that in cluster 2, as observed by principal component analysis (PCA) and t-distributed stochastic neighbor embedding (t-SNE) analysis (**Figures 5C, D**). To identify genes strongly correlated with cluster assignment based on GSE90074, we first generated a hierarchical clustering tree to remove 14 outlier samples, resulting in 79 remaining samples. After setting the soft-thresholding power (β) to 11, 10 gene modules were identified (**Figures 5E**; **Supplementary Figure S4**). Correlation analysis showed that the MEyellow module, comprising 643 genes, had the strongest correlation with cluster 1 and cluster 2 (**Figure 5F**). Therefore, the MEyellow module was selected for further analysis. This finding indicates that the biological processes represented by this module are closely associated with the fundamental heterogeneity among AS patients. We reasoned that genes within this module, which are central to AS pathophysiology, may harbor key drivers underlying intercluster differences.

Subsequently, baseline characteristics between the two clusters were compared to assess potential clinical relevance. As shown in **Table 2**, significant differences were observed in gender distribution (p = 0.018) and hyperlipidemia prevalence (p = 0.047) between clusters. Specifically, cluster 1 exhibited a higher proportion of females (52.5% vs. 28.3%, p = 0.018) and a lower prevalence of hyperlipidemia (70.0% vs. 86.8%, p = 0.047) than cluster 2. In addition, a significant difference was observed in CXCL5_rank levels (74.5 vs. 52.0, p = 0.031). No significant differences were identified for other clinical parameters, including ancestry, CAD_class, diabetes, hypertension, or BMI (p > 0.05).

**Table 2:** Clinical characteristics of the samples in different sub-clusters (* p < 0.05).

Characteristics	Total (n=93)	Cluster 1 (n=40)	Cluster 2 (n=53)	P-value
Gender, n(%)				0.018*
Female	36 (38.7%)	21 (52.5%)	15 (28.3%)	
Male	57 (61.3%)	19 (47.5%)	38 (71.7%)	
Ancestry, n(%)				0.083
AA	20 (21.5%)	12 (30.0%)	8 (15.1%)	
CAU	73 (78.5%)	28 (70.0%)	45 (84.9%)	
CAD_class, n(%)				0.678
2	31 (33.3%)	12 (30.0%)	19 (35.8%)	
3	26 (28.0%)	13 (32.5%)	13 (24.5%)	
4	36 (38.7%)	15 (37.5%)	21 (39.6%)	
Diabetes, n(%)				0.119
Yes	38 (40.9%)	20 (50.0%)	18 (34.0%)	
No	55 (59.1%)	20 (50.0%)	35 (66.0%)	
Hyperlipid, n(%)				0.047*
Yes	74 (79.6%)	28 (70.0%)	46 (86.8%)	
No	19 (20.4%)	12 (30.0%)	7 (13.2%)	
Hypertension, n(%)				0.262
Yes	82 (88.2%)	37 (92.5%)	45 (84.9%)	
No	11 (11.8%)	3 (7.5%)	8 (15.1%)	
CXCL5_rank, median(Q1, Q3)	61.0 (26.5,94)	74.5 (40.25,112.25)	52.0 (19,87)	0.031*
BMI, mean ± SD	28.97 ± 6.27	29.63 ± 7.53	28.47 ± 5.15	0.277

These results indicate that molecular subtyping based on the nine-gene signature stratifies AS patients into two subgroups with distinct clinical characteristics. The identification of these clusters supports the biological relevance of the selected feature genes and reveals clinically meaningful patient subgroups.

**Figure 6:**
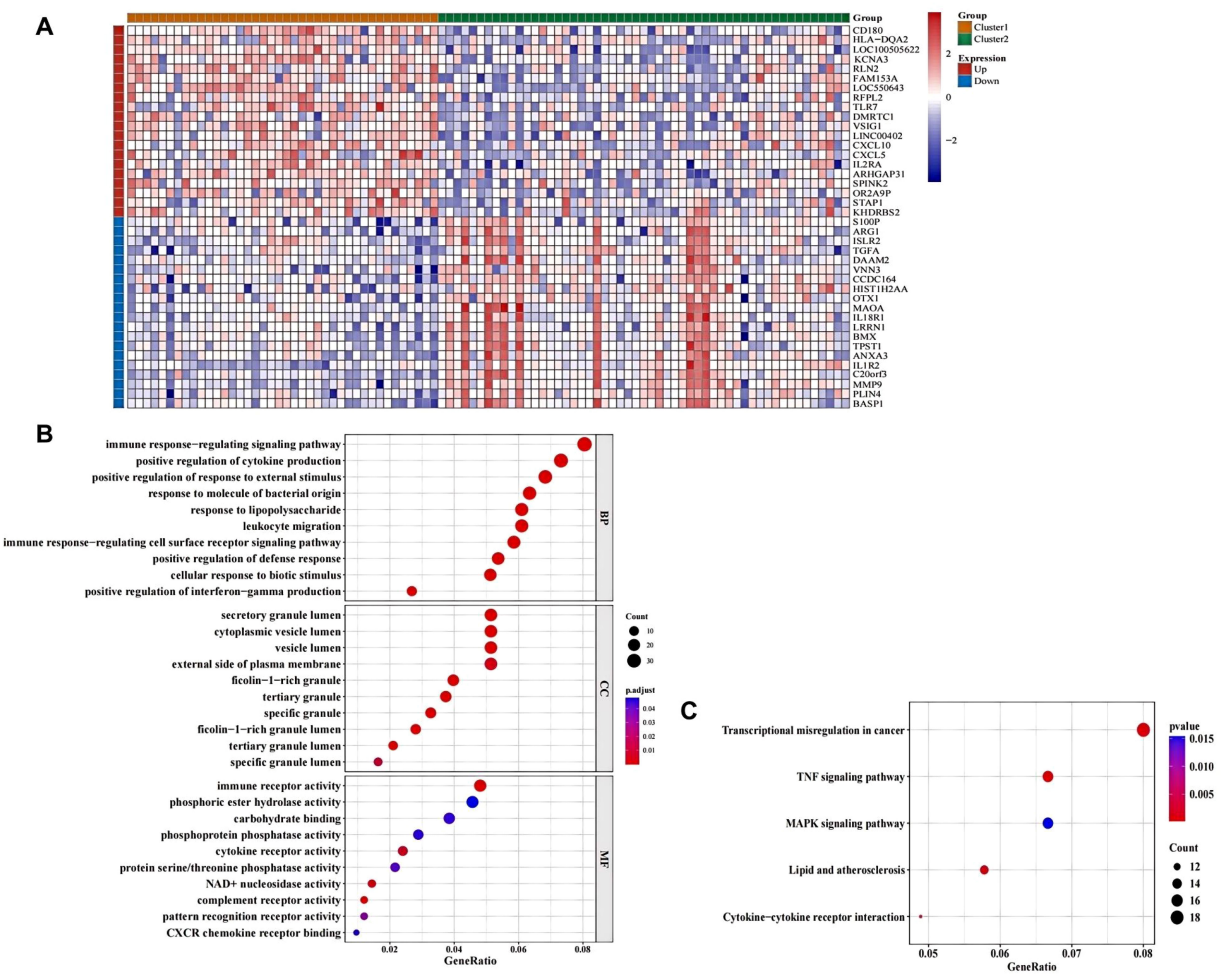
Analysis of DEGs between the different clusters. **(A)** Heatmap of 541 DEGs between cluster 1 and cluster 2 identified by differential analysis. **(B, C)** GO and KEGG enrichment analyses of the 541 DEGs.

To further explore intercluster heterogeneity, differentially expressed genes between cluster 1 and cluster 2 were identified. A total of 541 DEGs were detected, including 292 upregulated and 249 downregulated genes. The heatmap illustrates the top 10 genes with the highest levels of upregulation or downregulation (**Figure 6A**; **Supplementary Table S6**). Functional enrichment analysis showed that these DEGs were enriched in 145 GO biological process terms, such as immune response–regulating signaling pathways and positive regulation of cytokine production; 12 GO cellular component terms, including secretory granule lumen and cytoplasmic vesicle lumen; and 10 GO molecular function terms, such as immune receptor activity and phosphoric ester hydrolase activity (**Figure 6B**). In addition, five KEGG pathways—transcriptional misregulation in cancer, tumor necrosis factor (TNF) signaling pathway, mitogen-activated protein kinase (MAPK) signaling pathway, lipid and AS, and cytokine–cytokine receptor interaction—were significantly enriched (**Figure 6C**). Detailed enrichment results are provided in **Supplementary Tables S7**, **S8**.

To further clarify biological differences between the two clusters, KEGG pathway enrichment analysis was performed on cluster-specific DEGs (**Supplementary Tables S9**, **S10**). Focusing on pathways related to inflammation and hyperlipidemia, seven significantly upregulated pathways and one downregulated pathway were identified.

Genes upregulated in cluster 1 were predominantly enriched in immune and inflammatory pathways, including phagosome, Fc gamma R–mediated phagocytosis, chemokine signaling, neutrophil extracellular trap (NET) formation, and Toll-like receptor signaling pathways This coordinated enrichment underscored a pathogenesis centered on robust innate immune activation, neutrophil-driven inflammation, and sustained inflammatory responses, providing a molecular basis for the elevated CXCL5 levels observed clinically in this subgroup.

In contrast, cluster 2 showed downregulation of ribosomal protein–related pathways, suggesting alterations in fundamental cellular processes such as protein synthesis.

Collectively, these results indicate mechanistic divergence between the two clusters, with cluster 1 representing an immune–inflammatory–driven subtype and cluster 2 characterized by metabolic and ribosomal dysregulation, highlighting heterogeneity in AS pathogenesis.

### 3.5 Immune analysis of AS clusters

**Figure 7:**
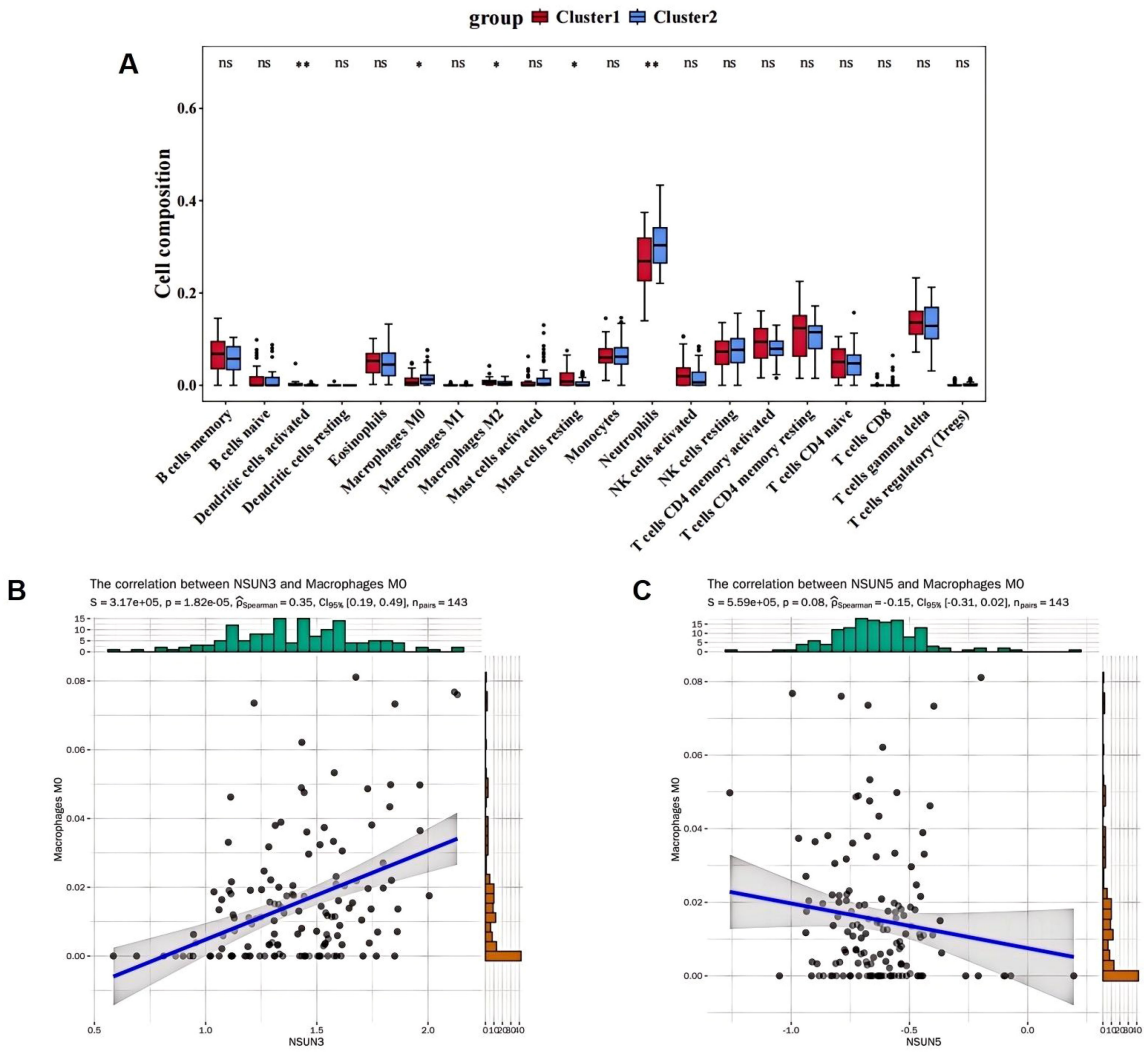
Immune analysis of different AS clusters. **(A)** Differences in immune cell subtypes between cluster 1 and cluster 2. **(B)** Correlation between NSUN3 and macrophages M0. **(C)** Correlation between NSUN5 and macrophages M0 (* p < 0.05, ** p < 0.01).

To investigate the immunological basis underlying molecular heterogeneity, immune cell proportions were inferred computationally. CIBERSORT analysis revealed significant differences in the relative abundances of five immune cell types, including activated dendritic cells, macrophages M0, macrophages M2, resting mast cells, and neutrophils, between cluster 1 and cluster 2 (**Figure 7A**). Next, correlations between immune cell levels and DE-MRs were evaluated. Both NSUN3 and NSUN5 showed the strongest correlations with macrophages M0 (R = 0.35 and R = −0.15, respectively) (**Figures 7B, C**).

### 3.6 Identification and validation of five biomarkers

**Figure 8:**
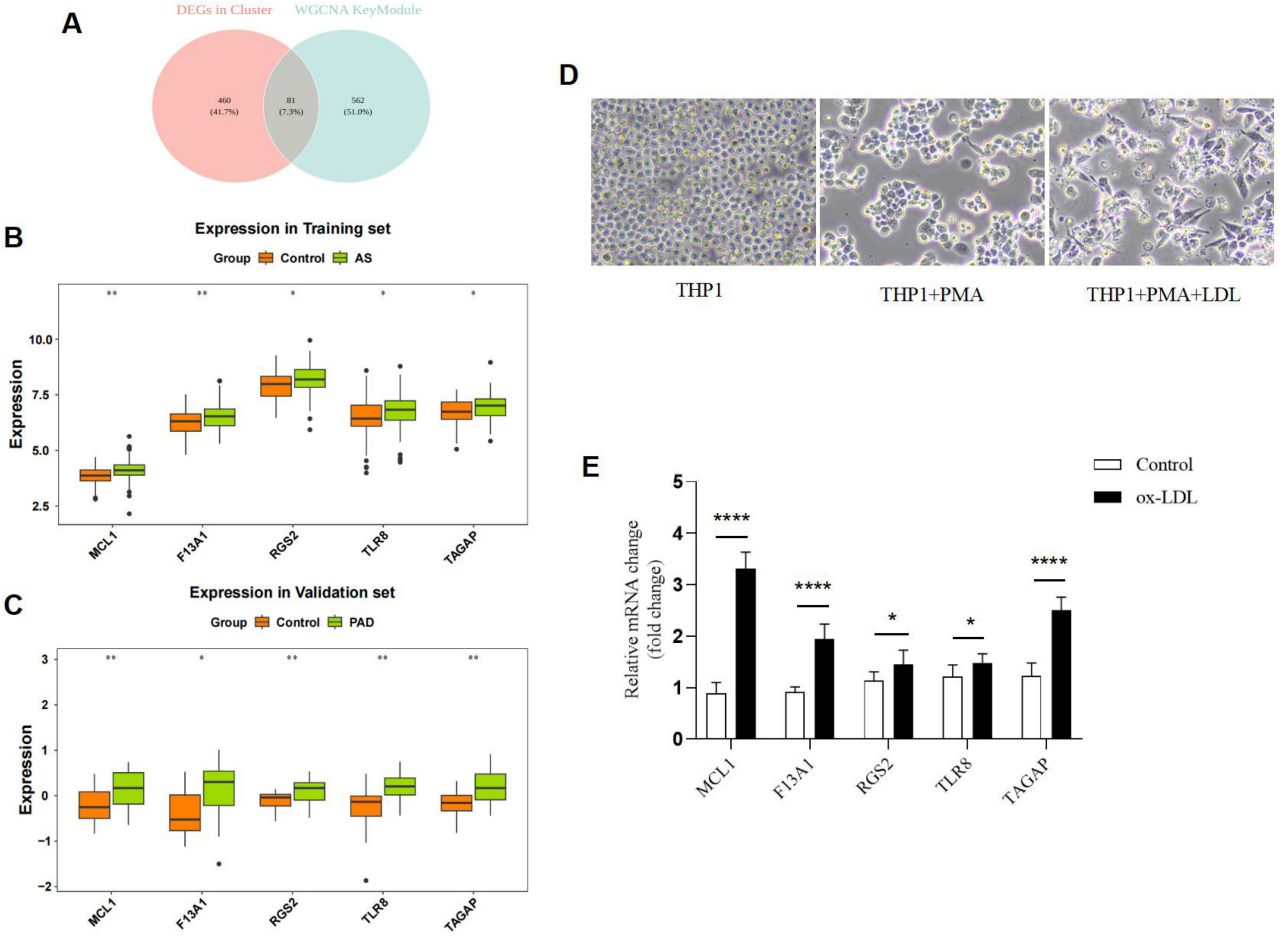
Identification and validation of five biomarkers in AS. **(A)** Venn diagram of 81 candidate biomarkers. **(B, C)** Expression trends of five biomarkers in the training and validation sets. **(D)** Construction of the foam cell model. **(E)** Verification of biomarkers expression in cell lines by qRT-PCR (*p < 0.05, ****p < 0.0001).

To pinpoint core biomarkers driving subtype differences, we identified 541 DEGs between cluster 1 and cluster 2. To refine this list, we prioritized genes that not only differed between clusters but also resided within the MEyellow co-expression network—the network most central to AS heterogeneity. We reasoned that intersecting the 541 DEGs with the 643 genes of the MEyellow WGCNA module would yield a high-confidence set of biomarkers that were both differentially regulated and integral to the key AS-related network (**Figure 8A**). Differential expression analysis between AS and control samples based on the training and validation sets identified five biomarkers—MCL1, F13A1, RGS2, TLR8, and TAGAP—that were significantly different between the two groups. Notably, expression trends of these biomarkers were highly consistent between the training and validation sets (**Figures 8B, C**).

To verify biomarker expression in AS, THP-1 cells were treated with oxidized low-density lipoprotein (ox-LDL) to generate macrophage-derived foam cells. qRT-PCR analysis revealed that expression levels of MCL1, F13A1, RGS2, TLR8, and TAGAP were significantly increased in the ox-LDL group (p < 0.05) (**Figures 8D, E**). Moreover, correlation heatmap analysis demonstrated strong correlations between feature genes and biomarkers (**Supplementary Figure S5**). Drug–gene interaction analysis identified 53 drugs, including aspirin and docetaxel, showing reciprocal associations with biomarkers including MCL1, F13A1, RGS2, and TLR8 (**Supplementary Figure S6**). Finally, a TF–mRNA–miRNA network comprising 14 transcription factors (TFs), four biomarkers, and 16 miRNAs was constructed. Notably, hsa-miR-20a-5p, hsa-miR-93-5p, hsa-miR-106b-5p, and hsa-miR-155-5p regulated two or more biomarkers, and 12 TFs directly regulated MCL1 (**Supplementary Figure S7**).

### 3.7 scRNA-seq analysis revealed the immune microenvironment of AS

**Figure 9:**
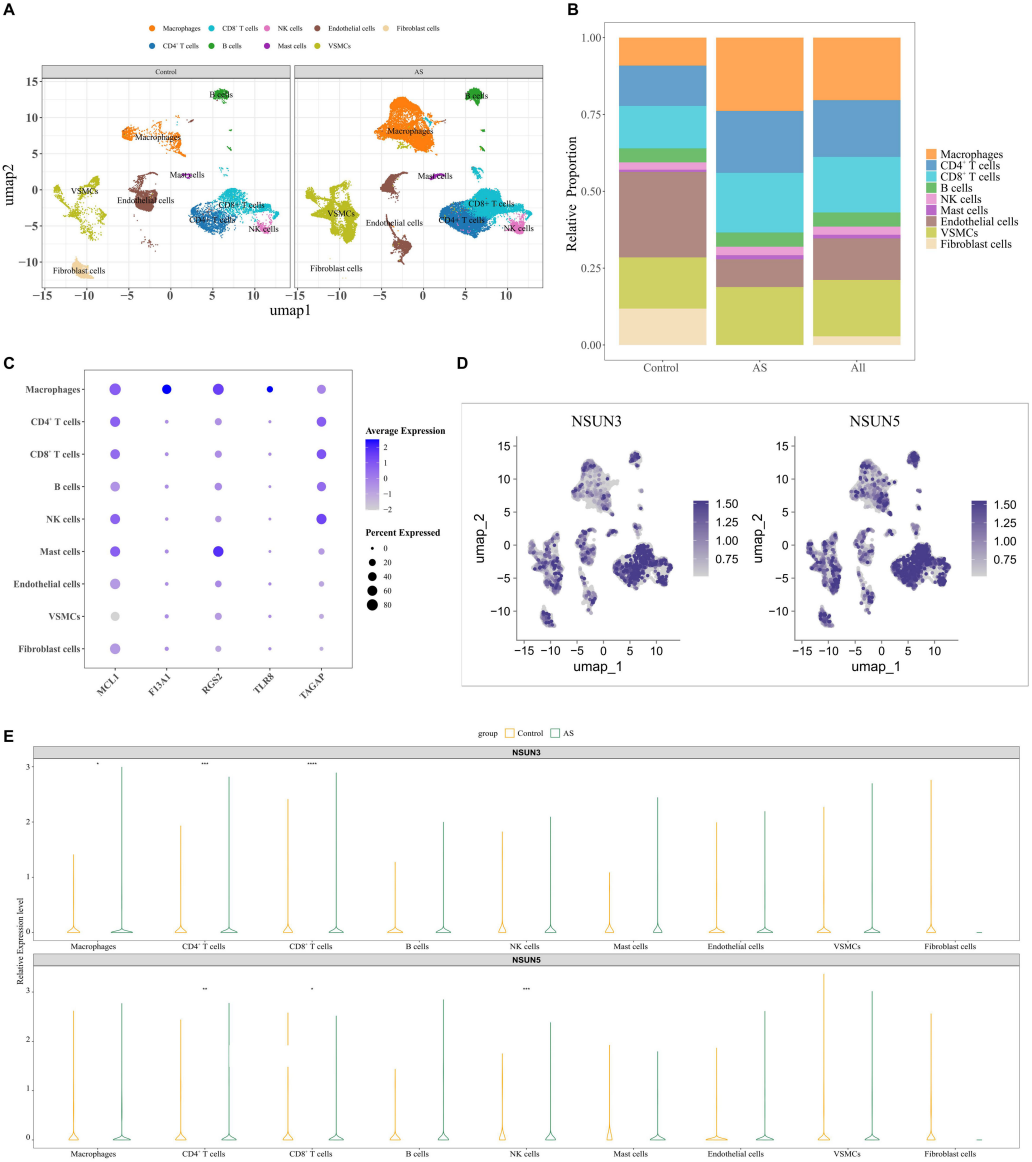
scRNA-seq analysis revealing the immune microenvironment of AS. **(A)** Distributions of cells across different samples visualized by UMAP dimensionality reduction. **(B)** Composition of different cell types in AS and control groups. **(C)** Expression level of biomarkers in different cell subtypes. **(D)** UMAP plots showing expression of two DE-MRs across cell subtypes in AS samples. **(E)** Expression levels of NSUN3 and NSUN5 across distinct cellular subpopulations between AS and control groups (* p < 0.05, ** p < 0.01, *** p < 0.001, **** p < 0.0001).

scRNA-seq analysis of tissue samples from the GSE159677 dataset was performed to resolve the cellular composition of the AS immune microenvironment and assess expression patterns of two DE-MRs across cell subsets. Following quality control, 2000 highly variable genes were selected for downstream analysis (**Figure 9A**). Principal component analysis indicated that 40 principal components captured the main biological variation and were used for clustering. UMAP analysis identified 23 distinct cell subpopulations, which were annotated into nine major cell types: macrophages, CD4^-^ T cells, CD8^-^ T cells, B cells, natural killer (NK) cells, mast cells, endothelial cells, vascular smooth muscle cells (VSMCs), and fibroblasts (**Figure 9B**). These results clearly delineated the immune cell landscape in the carotid atherosclerotic plaques. The five biomarkers showed high expression in macrophages (**Figure 9C**), suggesting a key role for macrophages in AS pathogenesis. Further analysis showed that NSUN3 expression was significantly increased in AS samples compared with control samples (p < 0.05) (**Figures 9D, E**).

### 3.8 The expression levels of NSUN3 and NSUN5 in the foam cell model

**Figure 10:**
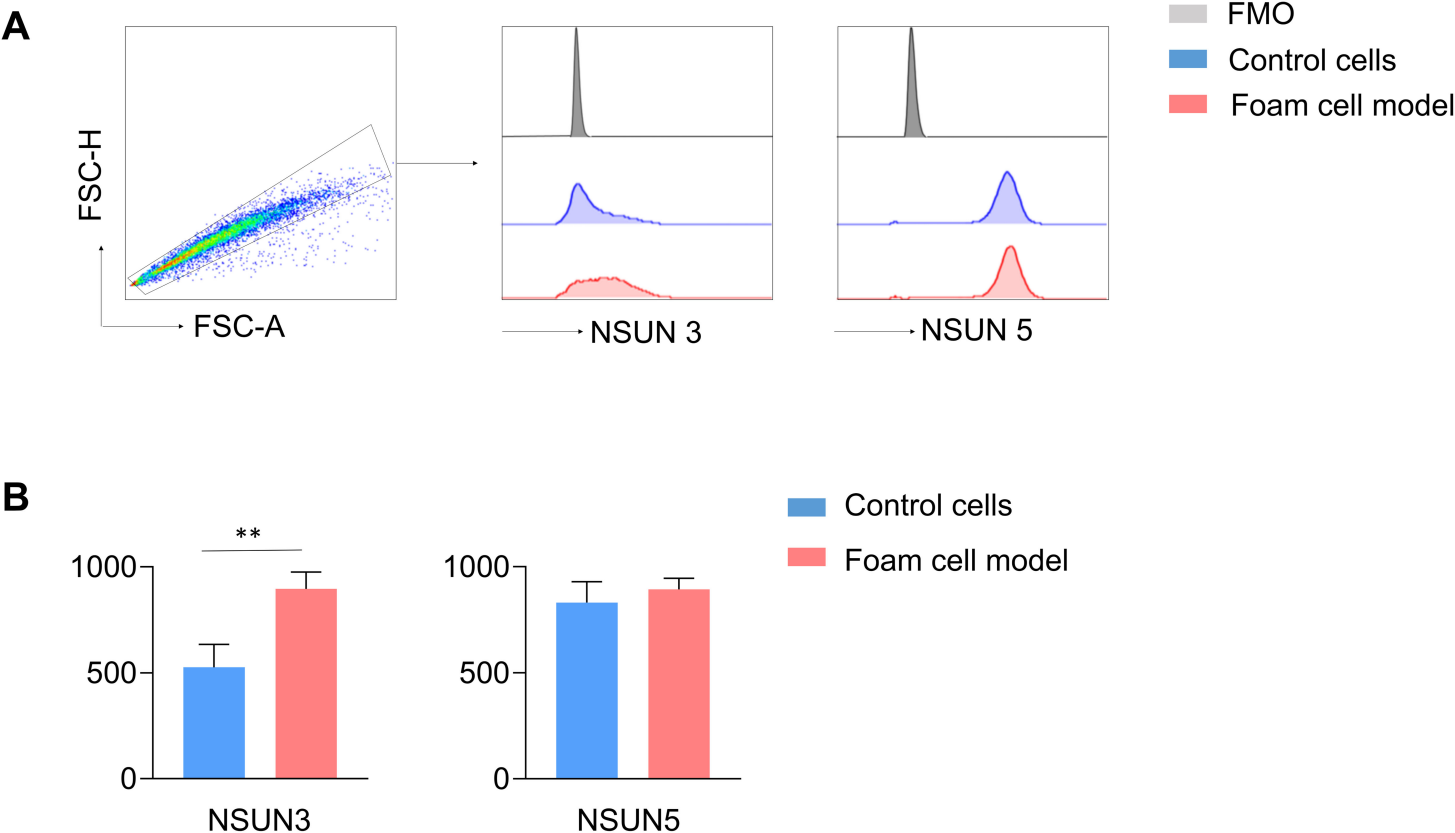
The expression levels of NSUN3 and NSUN5 in the foam cell model. **(A)** MFI histograms for NSUN3 and NSUN5 in the foam cell model. **(B)** The expression levels of NSUN3 and NSUN5 in the foam cell model. n=4/group (** p < 0.01).

The relative exprssion level (measured by mean fluorescence intensity, MFI) of NSUN3 was statistically increased in the foam cell group (896.50±79.29) compared with the control cell group (526.50±107.28) (p < 0.05), whereas no significant change was observed in NSUN5 (832.25±98.05) vs. (894.00±52.33) (p > 0.05) (**Figures 10A, B**).

### 3.9 The immune and inflammatory effects of NSUN3 in macrophages during AS progression

**Figure 11:**
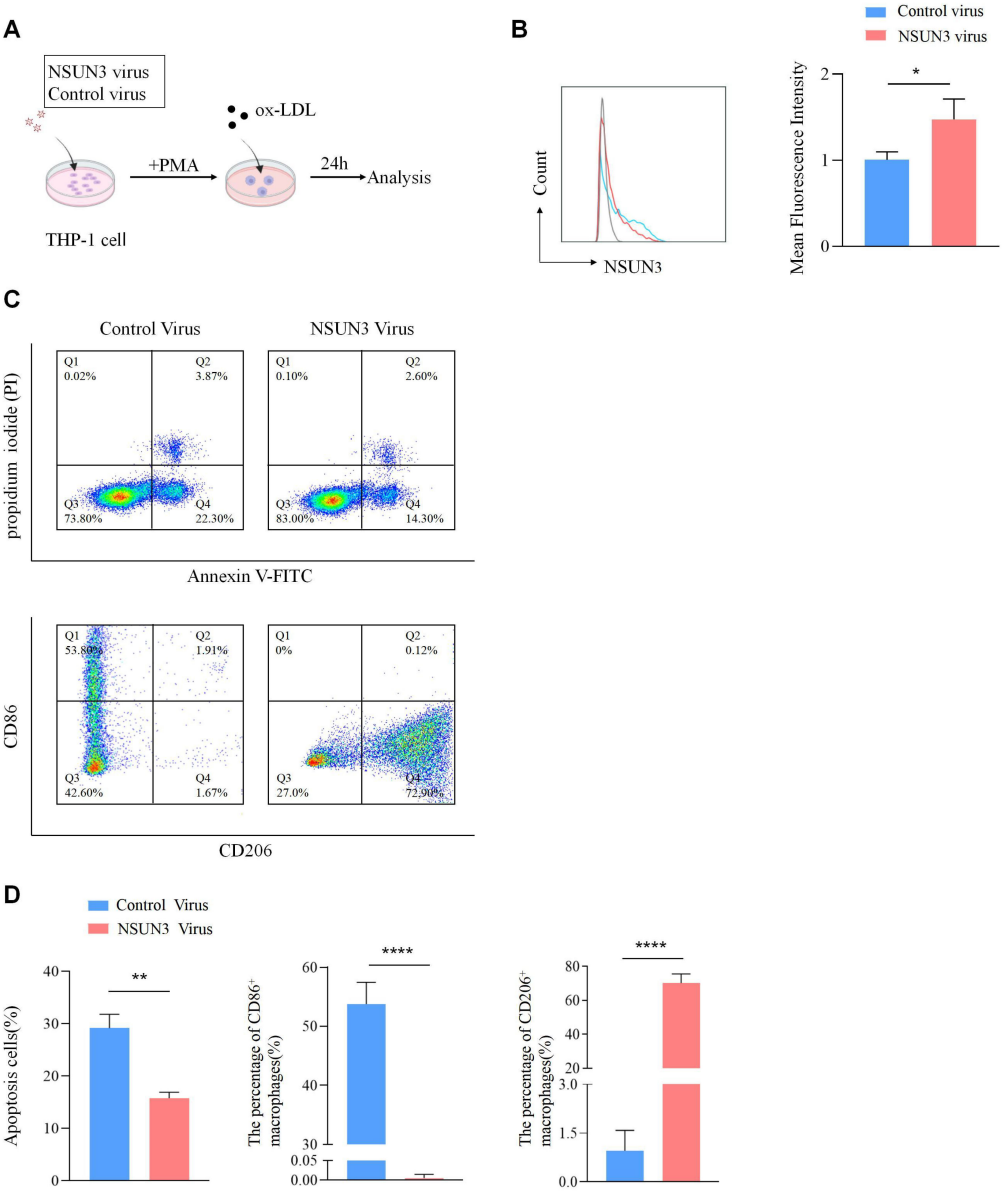
NSUN3 transfection inhibits apoptosis and promotes M1 to M2 polarization in the AS model. **(A)** Schematic illustrating the experimental procedure for THP-1 cell treatment. THP-1 cells were infected with control virus or NSUN3 virus for 3 days, followed by PMA-induced differentiation into M0 macrophages for 48 h and ox-LDL treatment for 24 h to generate foam cells. Cells were then harvested for flow cytometry analysis. **(B)** NSUN3 expression efficiency in ox-LDL–treated THP-1 cells under the indicated conditions (n = 3/group). **(C, D)** Annexin V/PI apoptosis analysis and CD86^-^/CD206^-^ macrophage polarization in NSUN3-transfected cells and controls. n=3/group (*p < 0.05, **p < 0.01, ****p < 0.0001).

**Figure 12:**
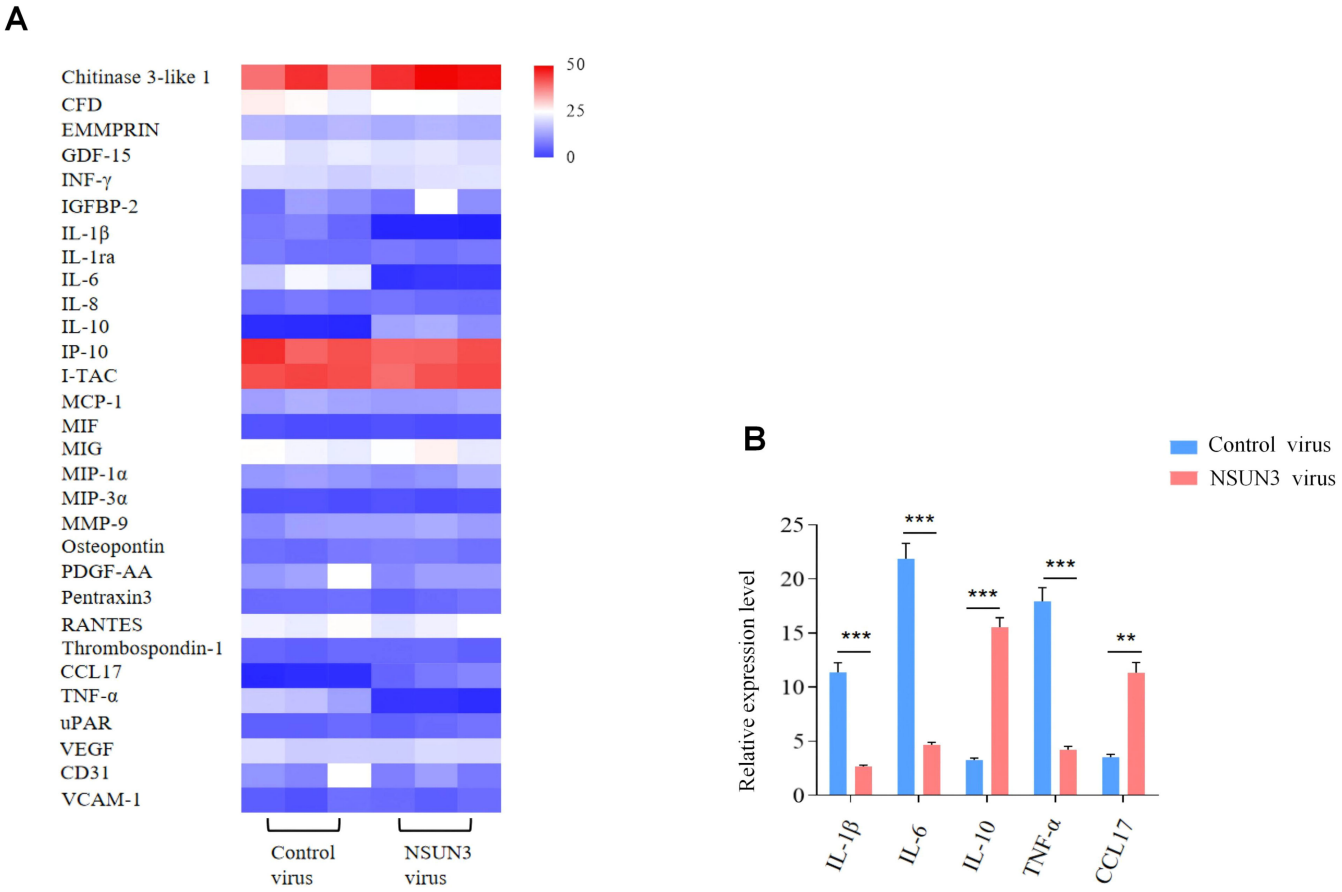
Cytokine profiling of the NSUN3 transfected cells and controls. **(A)** Heatmap showing relative pixel intensity of cytokine spots in the NSUN3-transfected model and controls. **(B)** Bar graphs showing the most significantly dysregulated cytokines (**p < 0.01, ***p < 0.001).

To investigate the functional role of NSUN3, THP-1 macrophages were transfected with an NSUN3-overexpressing vector or a control vector, followed by ox-LDL treatment to establish a foam cell model (**Figures 11A, B**). Flow cytometry analysis showed that the apoptosis rate in the NSUN3 vector group (15.79% ± 1.12%) was significantly lower than that in the control vector group (29.17% ± 2.63%) (independent Student’s *t*-test, n=3, p = 0.001). In addition, the proportion of proinflammatory (M1) macrophages indicated by CD86^+^ was significantly reduced, whereas the proportion of anti-inflammatory (M2) macrophages indicated by CD206^+^ was markedly increased in the NSUN3 vector group compared with the control group (**Figures 11C, D**). Cytokine profiling using a Proteome Profiler Human XL Cytokine Array demonstrated that NSUN3 overexpression significantly increased anti-inflammatory cytokines, including interleukin (IL)-10 and C–C motif chemokine ligand (CCL)17, while decreasing proinflammatory cytokines, including IL-1β, IL-6, and tumor necrosis factor α (TNF-α) (**Figures 12A, B**; **Supplementary Figure S8**).

### 3.10 Construction of the ceRNA network of biomarkers

**Figure 13:**
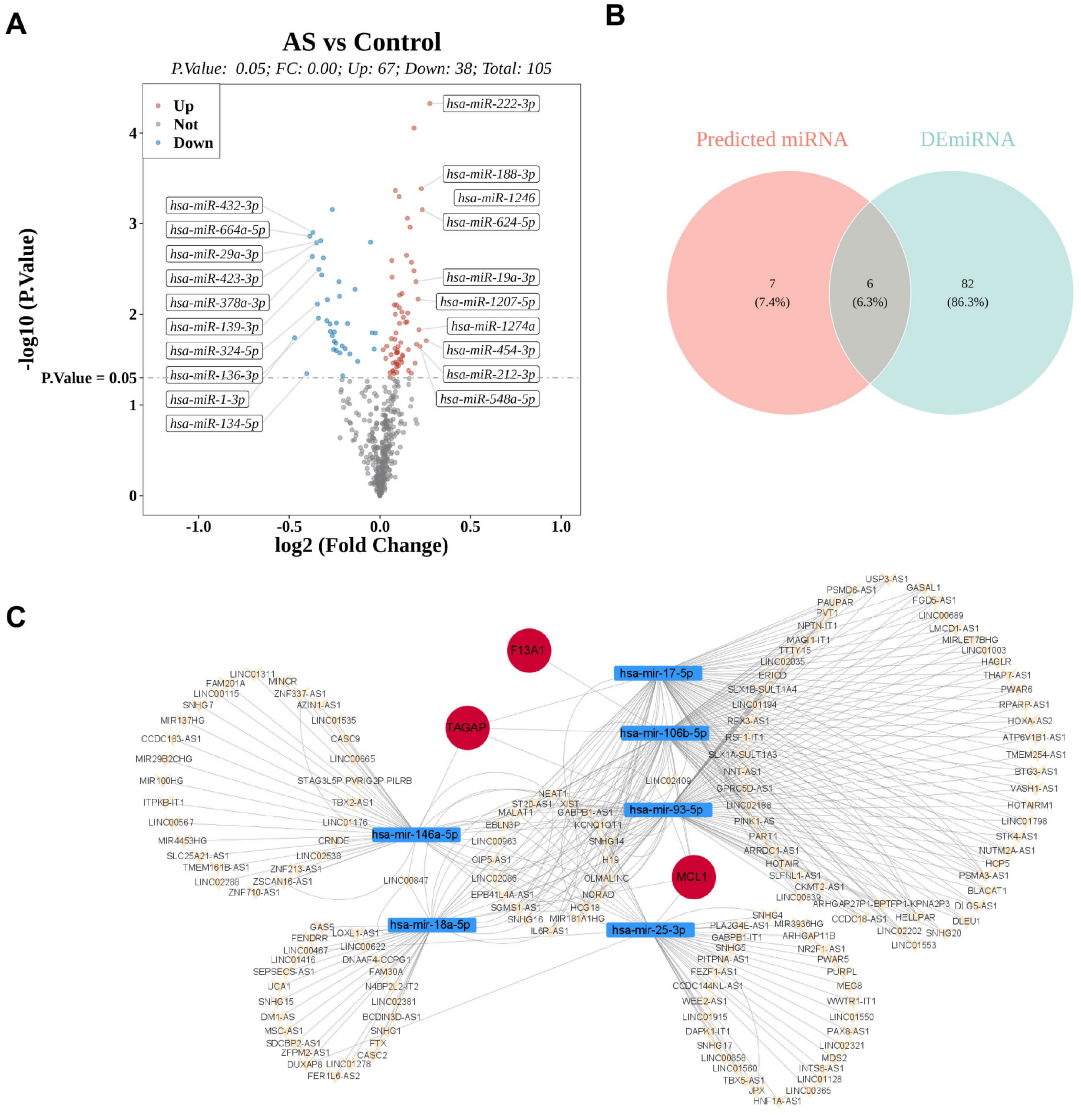
Construction of the ceRNA network of biomarkers. **(A)** Volcano plot of DE-miRNAs between AS and control samples in GSE59421 and those identified in the TF–mRNA–miRNA network. **(B)** Venn diagram showing six mature miRNAs.

A total of 105 differentially expressed miRNAs (DE-miRNAs) were identified between AS cases (AS = 33) and controls (control = 63) in GSE59421, including 67 upregulated and 38 downregulated miRNAs (**Supplementary Table S11**). These DE-miRNAs were intersected with miRNAs identified from the TF–mRNA–miRNA network and visualized using a volcano plot (**Figure 13A**). After deduplication, 88 DE-miRNAs were obtained and further intersected with 13 miRNAs from the miRNet database, resulting in six mature miRNAs (**Figure 13B**). These mature miRNAs corresponded to seven miRNAs—hsa-miR-17-5p, hsa-miR-17-3p, hsa-miR-93-5p, hsa-miR-106b-5p, hsa-miR-18a-5p, hsa-miR-25-3p, and hsa-miR-146a-5p—in the TF–mRNA–miRNA network and were used to construct the ceRNA network. Among the identified regulatory axes, lncRNA NEAT1/hsa-miR-17-5p/MCL1 was highlighted (**Figure 13C**).

### 3.11 Clinical relevance of the five biomarkers

To assess the clinical relevance of the five biomarkers (MCL1, F13A1, RGS2, TLR8, and TAGAP), we analyzed associations between their expression levels and key clinical parameters of AS. Based on baseline characteristics of the GSE90074 dataset (**Table 1**), four parameters significantly associated with disease status were identified: gender, hyperlipidemia, disease severity (CAD_class), and the inflammatory marker CXCL5.

Spearman correlation analysis within AS patients showed that RGS2, TLR8, and TAGAP expression levels were negatively correlated with CXCL5 (p < 0.05) (**Supplementary Table S12**). Stratification by disease status and hyperlipidemia revealed that MCL1, F13A1, TLR8, and TAGAP were significantly upregulated in patients with both AS and hyperlipidemia compared with healthy individuals without hyperlipidemia (p < 0.05) (**Supplementary Table S13**). This indicated that the upregulation of these genes was closely coupled with the high-risk clinical phenotype of “disease + key risk factor”, demonstrating their potential for patient risk stratification.

In terms of disease severity, stratification analysis by coronary artery disease severity indicated a direct correspondence between gene expression levels and the degree of anatomical lesions (**Supplementary Table S14**). Among them, MCL1, F13A1, and TAGAP were significantly upregulated early in the disease (CAD_class 2) (p < 0.05), suggesting their value as early diagnostic markers, while RGS2 and TLR8 showed the highest expression at the most severe disease stage (CAD_class 4) (p < 0.05), indicating their association with disease progression.

From the perspective of demographic characteristics, stratification analysis by gender and disease status revealed specific patterns of gene expression (**Supplementary Table S15**). Several genes (e.g., F13A1, TLR8, TAGAP) showed significantly lower expression in Control Males compared to other groups (p < 0.05), while TLR8 exhibited significant baseline gender differences even in the healthy population (p < 0.05Overall, these findings indicate that the five biomarkers are associated not only with AS presence but also with inflammation, risk stratification, disease progression, and gender-specific characteristics, supporting their potential clinical utility.

## 4 Discussion

To date, over 170 known RNA chemical modifications have been identified, with methylation modifications accounting for more than 60% of these, including 6-methyladenosine (m6A) and 5-methylcytosine (m5C) (20, 21). A growing body of research has highlighted the crucial role of methylation in AS and its associated immune regulation (22). The m6A “writer” methyltransferase METTL3 has been reported as a key hub in hemodynamic forces. Oscillatory stress in AS stimulated the upregulation of METTL3 expression, which led to attenuated RelA/p65 phosphorylation, subsequently augmenting nuclear factor kappa B (NF-κB) activity, exacerbating inflammatory responses, and facilitating plaque formation. Increased METTL3 abundance also regulated the levels of nucleotide-binding domain leucine-rich repeat protein 1 (NLRP1) and Kruppel-like factor 4 (KLF4), inducing endothelial inflammation and arterial expansion (23). The RNA-binding protein Matrin-3 exerted anti-inflammatory effects in macrophages by orchestrating the formation of the METTL3–METTL4 methyltransferase complex to promote m6A-dependent mRNA degradation, thereby attenuating ox-LDL-triggered mitogen-activated protein kinase (MAPK) signaling activation and subsequent inflammatory responses (24). Luo et al. showed that NSUN2 methylated ICAM-1 mRNA and increased leukocyte adhesion to vascular endothelial cells, while ICAM-1 expression was significantly diminished in NSUN2^-^/^-^ rats. Mechanistically, TNF-α or homocysteine increased NSUN2 methyltransferase activity by inhibiting NSUN2 phosphorylation. Moreover, donor NSUN2 deficiency hindered the development of allograft arteriosclerosis *in vivo* (3). In addition, NSUN2 was reported to regulate ALYREF nuclear–cytoplasmic trafficking efficiency, directly influencing cytoplasmic accumulation of m5C-modified mRNAs involved in leukocyte recruitment and vascular smooth muscle cell apoptosis. Mechanistically, NSUN2-mediated m5C modification stabilized ALYREF–mRNA complexes, amplifying inflammatory signaling cascades through enhanced translation fidelity (25). However, diagnostic biomarkers and immune regulatory functions of m5C regulators in AS remain unclear. In this study, we screened AS-related genes significantly correlated with m5C regulators, identified potential biomarkers, constructed a ceRNA network, and conducted a series of experiments to analyze immune and inflammatory effects of m5C regulators on macrophage function. These findings may provide novel insights into therapeutic strategies for AS.

Previously, Nakano et al. reported that knockout of the RNA methyltransferase NSUN3 resulted in marked reductions in mitochondrial protein synthesis and oxygen consumption, leading to impaired mitochondrial activity (26). They demonstrated that the biogenesis pathway of the f5C34 modification in mitochondrial transfer RNA^met^ (mt-tRNA ^met^) begins with NSUN3-mediated catalytic activity. Quantitative analysis of the human mitochondrial proteome revealed a striking codon adaptation: among the 13 mtDNA-encoded polypeptides (containing a total of 207 methionine incorporation sites), AUA codons (167 loci) were 4.2-fold more prevalent than canonical AUG codons (40 loci). This evolutionary adaptation established f5C-dependent AUA decoding as a critical quality control checkpoint. NSUN3 depletion caused failure of wobble base-pair resolution at AUA codons, resulting in translation stalling and collapse of mitochondrial proteome integrity. Mitochondrial dysfunction is recognized as an essential factor in AS initiation and progression. For example, serine/arginine-rich splicing factor 3 (SRSF3) maintained effective clearance of oxidized lipids and apoptotic debris in AS lesions by regulating mitochondrial proteostasis (27–29). Phenotypic consequences of NSUN5 deficiency in mammalian cells include reduced cell proliferation and size, highlighting the importance of m5C regulators in ribosome function and normal cellular physiology (30). In the present study, NSUN3 and NSUN5 were identified as two DE-MRs between AS and healthy control samples. Hub genes in the constructed PPI network included multiple ribosomal proteins (e.g., RPL12, RPL15, RPL18A). As core sensors of cellular stress, alterations in ribosomal function can integrate multiple pathological processes in AS, including endoplasmic reticulum stress, metabolic dysregulation, and inflammatory signaling (31). These findings extend the role of m5C modifications beyond protein synthesis, suggesting broader involvement in the AS pathological environment through regulation of ribosomal function (32).

Atherosclerosis is a chronic inflammatory disease of the arterial vessel wall, with prominent involvement of immune cells, including monocytes, macrophages, dendritic cells (DCs), T cells, and B cells (18, 33, 34). AS is initiated by endothelial dysfunction, which promotes secretion of chemotactic mediators such as CCL2 and chemokine (C-X-C motif) ligand 1 (CXCL1), recruiting circulating myeloid cells, particularly monocytes and neutrophils. Monocytes infiltrate lesions and differentiate into macrophages, which uptake lipoproteins and form foam cells that constitute the central core of atheromas. Neutrophils act as early responders, releasing reactive oxygen species via NADPH oxidase and myeloperoxidase, modifying leukocyte adhesion and promoting endothelial dysfunction. In addition, neutrophils secrete granule proteins that act as extracellular inflammatory mediators, with growing evidence supporting a role for neutrophil extracellular traps (NETs) (35–37). DCs characterized by the CD11c^-^ human leukocyte antigen-DR (HLA-DR^-^) phenotype function as professional antigen-presenting cells via major histocompatibility complex class II (MHC-II)/T cell receptor (TCR) interactions, driving naive T cell activation. In AS, DC subsets (e.g., CD103^-^ vs. CD8α^-^) differentially regulate disease progression through scavenger receptor–mediated uptake of oxidized LDL, antigen presentation, and interleukin (IL)-23–driven T helper 17 (Th17) polarization (38, 39). Mast cells, predominantly localized in the arterial adventitia, promote extracellular matrix degradation through proteolytic enzymes targeting elastic fibers and laminae, thereby contributing to vascular injury and maladaptive remodeling (40). These observations are consistent with our enrichment and immune analyses of MRRGs and AS clusters.

Our CTD analysis revealed that hub MRRGs, including TLR4 and TP53, exhibited higher inference scores. The TLR family comprises pattern-recognition receptors located on the cell membrane. Preclinical studies have shown that inhibition of the TLR4/MyD88/NF-κB axis attenuates neointimal hyperplasia and plaque destabilization (41). Mechanistically, ox-LDL induces TLR4/NF-κB–dependent transcription of matrix metalloproteinase 9 (MMP-9), promoting extracellular matrix degradation and fibrous cap thinning. TLR4 also regulates macrophage polarization; lipopolysaccharide (LPS)–TLR4 interaction promotes M1 polarization and increases proinflammatory cytokines, including TNF-α, IL-1β, IL-6, and monocyte chemoattractant protein 1 (MCP-1) (42). TP53 is a stress-responsive transcription factor activated following genomic damage. TP53-inducible enzymes regulate cellular redox homeostasis and protect against apoptosis by scavenging reactive oxygen species (ROS). These antioxidant functions mitigate oxidative stress–induced DNA damage and genomic instability, promoting cell survival under stress. Increasing evidence suggests that TP53-mediated prosurvival mechanisms contribute to AS pathophysiology through modulation of oxidative stress responses in vascular cells (43, 44).

We constructed a diagnostic prediction model for AS using the LASSO regression algorithm and analyzed regulatory mechanisms of feature genes through a TF–mRNA–miRNA network. PRDM1, regulated by five miRNAs (hsa-miR-223-3p, hsa-miR-181a-5p, hsa-miR-20a-3p, hsa-miR-99a-5p, and hsa-miR-20a-5p) and three TFs (CEBPB, PAX5, and STAT3), may have therapeutic relevance in AS. These findings are consistent with the result reported by Folgado et al., who demonstrated that PRDM1fl/fl Aicda-Cre^+^/ki Ldlr^−/−^ chimeras increased AS via germinal center-derived plasma cells and/or antibodies (45).

Based on feature gene analysis, AS samples were divided into clusters, with significant differences observed in five immune cell types: activated DCs, macrophages M0, macrophages M2, resting mast cells, and neutrophils. NSUN3 showed the strongest correlation with macrophages M0, which was further validated by flow cytometry. We found that the relative level of NSUN3 was significantly increased in PBMC-derived foam cells compared with the control cells. The apoptosis cells were significantly reduced in NSUN3-transfected macrophages. And the NSUN3 infection could stimulate the transition of foam cells from M1 to M2. Macrophages M0 are the undifferentiated cell type that can be guided to polarized cell types, according to corresponding signals. The switch between polarized macrophages, for instance, from M2 to M1, determined the transformation of the inflammatory microenvironment in the progression of AS. The classically activated M1 subset orchestrated inflammatory pathogenesis and mediated tissue degradation cascades; in contrast, the alternatively activated M2 compartment executed reparative functions, modulated plaque biomechanical stability and repressed pro-inflammatory signaling networks. The knockdown of the m5C regulator NSUN3 has been shown to increase the infiltration of M1 macrophages and decrease the level of M2 macrophages in xenograft models (46). However, the role of NSUN5 in macrophage function remains poorly understood and warrants further investigation.We identified five biomarkers, namely MCL1, F13A1, RGS2, TLR8, and TAGAP, for AS and investigated their expression levels in AS cell lines. Fontaine et al. previously reported that myeloid deletion of MCL-1 promoted apoptosis and lipid accumulation in AS plaques, implicating MCL-1 in the survival and differentiation of leukocytes, particularly neutrophils (47). The coagulation factor XIII A gene (F13A1) is a recognized M2 anti-inflammatory macrophage marker and can be regulated by neuron-derived orphan receptor 1, which is expressed in human AS lesions (48). Genetic polymorphisms in RGS2 have been associated with intima–media thickening of the carotid artery in both hypertensive and general populations, indicating a potential role in AS pathogenesis (49). In addition, Allen et al. demonstrated that TLR8 activation led to enrichment of microbial small RNA (msRNA) on LDL and promoted pro-inflammatory macrophage polarization (50), highlighting the role of TLR8 in AS. However, the potential effects of TAGAP in AS have not yet been fully investigated.

This study established the clinical relevance of five potential biomarkers in AS through systematic correlation analyses with well-established clinical parameters. Our findings demonstrated that these genes were significantly associated with key aspects of AS pathology, including inflammatory mechanisms, hyperlipidemia interaction, disease severity progression, and gender-specific effects. The biomarkers showed distinct clinical utility patterns: MCL1, F13A1, and TAGAP served as early-stage markers, whereas RGS2 and TLR8 were associated with advanced disease, providing complementary information for disease staging. Furthermore, the gender-specific expression patterns, particularly for TLR8, highlighted the importance of considering sex differences in AS biomarker research. Rather than replacing existing diagnostic methods, these transcriptomic biomarkers provide added value by offering molecular-level insights into disease mechanisms, enabling risk stratification, and facilitating early detection.

Chang et al. reported that genetic silencing of MCL-1 augmented aspirin-induced viability loss and apoptosis in glioma cells (51). Higher expression of the hematopoietic transcription factor RUNX1 target F13A1 was associated with acute events in patients with cardiovascular disease receiving aspirin or ticagrelor (52). These findings are consistent with our results showing that aspirin was correlated with both MCL-1 and F13A1. Moreover, we constructed a ceRNA network by identifying six mature miRNAs corresponding to seven miRNAs in the TF–mRNA–miRNA network. We found that lncRNA NEAT1 could regulate MCL-1 expression by targeting hsa-miR-17-5p. Previous studies have shown that NEAT1 regulates the protein kinase B (AKT)/mammalian target of rapamycin (mTOR) signaling pathway in ox-LDL–induced human aortic endothelial cells, accelerating proliferation and inhibiting apoptosis, suggesting that NEAT1 may be a potential therapeutic target in AS (53). Furthermore, reduced levels of miR-17-5p and enhanced Beclin-1–mediated autophagy increased cholesterol efflux from THP-1 macrophage-derived foam cells (54). Downregulation of NEAT1 expression has also been shown to inhibit proliferation and induce apoptosis in rheumatoid arthritis fibroblast-like synoviocytes by targeting miR-17-5p and inhibiting STAT3 (55). However, the mechanisms underlying the lncRNA NEAT1/miR-17-5p/MCL-1 axis in AS require further investigation.

Several limitations of this study should be acknowledged. First, the number of samples available in public databases for both AS and healthy controls was relatively small, which may introduce bias and limit the generalizability of the findings. Prospective validation in longitudinal cohorts with documented cardiovascular outcomes will be essential to establish prognostic value. Second, additional functional studies are required to substantiate the diagnostic and therapeutic relevance of the identified biomarkers and their associated biological pathways, which may guide the development of targeted therapies. Third, the molecular mechanisms of the identified ceRNA networks, such as the lncRNA NEAT1/miR-17-5p/MCL-1 axis, in AS progression remain incompletely understood. Thus, more comprehensive studies are needed to clarify these limitations and provide a more accurate understanding of the molecular mechanisms underlying AS.

## 5 Conclusions

In conclusion, our findings revealed significant differences in the expression of m5C regulators between AS and healthy control samples, which were used to identify MRRGs and construct a diagnostic model for AS. We further identified five potential diagnostic biomarkers—MCL1, F13A1, RGS2, TLR8, and TAGAP. In addition, our study demonstrated the immune and inflammatory effects of NSUN3 in AS, providing new evidence that may inform the development of novel immunotherapeutic strategies in clinical practice.

## Data Availability

The datasets presented in this study can be found in online repositories. The names of the repository/repositories and accession number(s) can be found in the article/**Supplementary Material**.
