# Supplementary material for: The role of 5-methylcytosine regulator-related genes in diagnostic and immune regulatory functions in atherosclerosis
Source: Front Immunol. 2026 Jan 9;16:1636323. doi: 10.3389/fimmu.2025.1636323 (PMC12827604; doi:10.3389/fimmu.2025.1636323)
Supplement: Supplementary file 1 [file DataSheet1.zip › Suppelementary files-/Supplement Figures/S5.pdf]

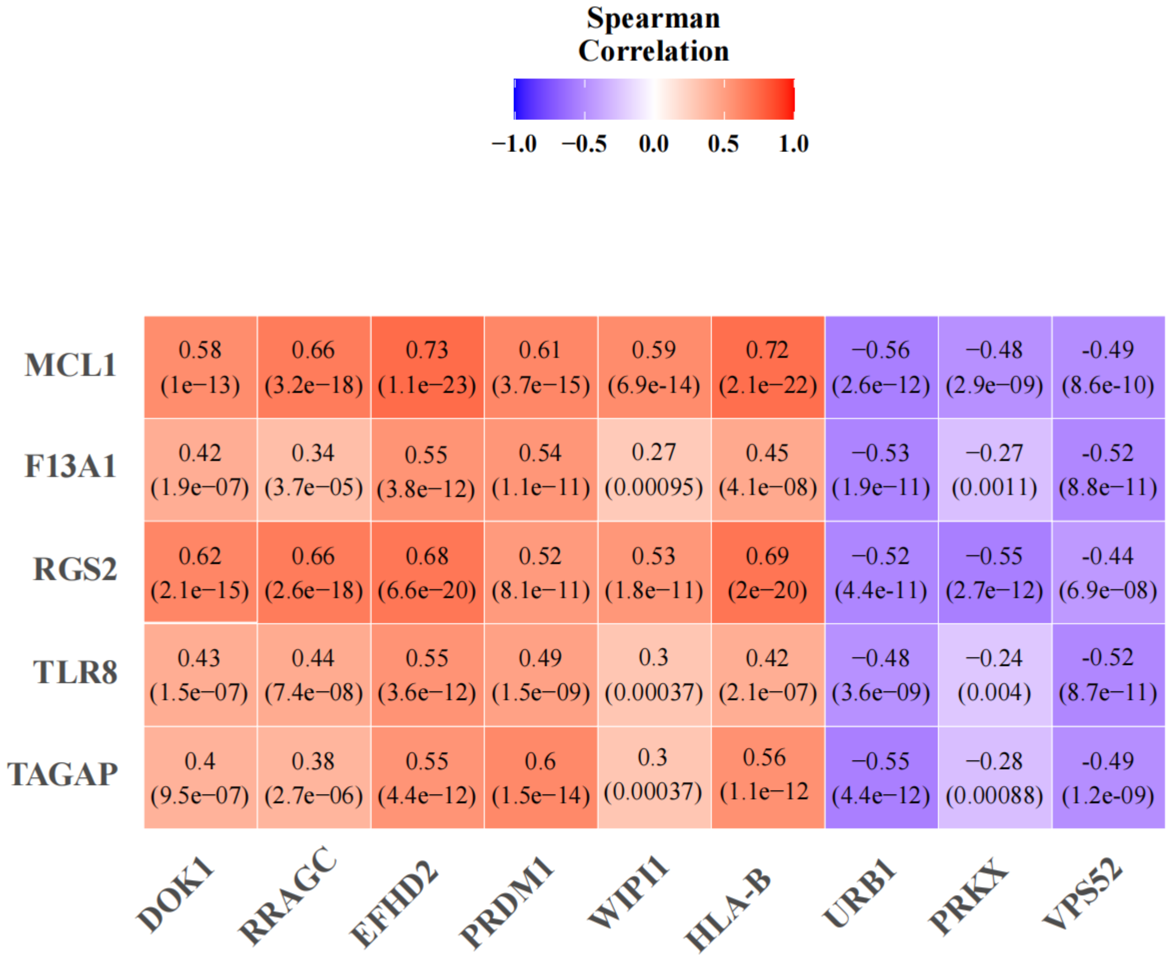
Spearman
Correlation
−1.0
−0.5
0.0
0.5
1.0
MCL1
0.58 (1e−13)
0.66 (3.2e−18)
0.73 (1.1e−23)
0.61 (3.7e−15)
0.59 (6.9e-14)
0.72 (2.1e−22)
−0.56 (2.6e−12)
−0.48 (2.9e−09)
-0.49 (8.6e-10)
F13A1
0.42 (1.9e−07)
0.34 (3.7e−05)
0.55 (3.8e−12)
0.54 (1.1e−11)
0.27 (0.00095)
0.45 (4.1e−08)
−0.53 (1.9e−11)
−0.27 (0.0011)
-0.52 (8.8e−11)
RGS2
0.62 (2.1e−15)
0.66 (2.6e−18)
0.68 (6.6e−20)
0.52 (8.1e−11)
0.53 (1.8e−11)
0.69 (2e−20)
−0.52 (4.4e-11)
−0.55 (2.7e−12)
-0.44 (6.9e−08)
TLR8
0.43 (1.5e−07)
0.44 (7.4e−08)
0.55 (3.6e−12)
0.49 (1.5e−09)
0.3 (0.00037)
0.42 (2.1e−07)
−0.48 (3.6e−09)
−0.24 (0.004)
-0.52 (8.7e−11)
TAGAP
0.4 (9.5e−07)
0.38 (2.7e−06)
0.55 (4.4e−12)
0.6 (1.5e−14)
0.3 (0.00037)
0.56 (1.1e−12
−0.55 (4.4e−12)
−0.28 (0.00088)
-0.49 (1.2e-09)
DOK1
RRAGC
EFHD2
PRDM1
WIPI1
HLA-B
URB1
PRKX
VPS52
