# Supplementary figures and images for: The role of 5-methylcytosine regulator-related genes in diagnostic and immune regulatory functions in atherosclerosis
Source: Front Immunol. 2026 Jan 9;16:1636323. doi: 10.3389/fimmu.2025.1636323 (PMC12827604; doi:10.3389/fimmu.2025.1636323)

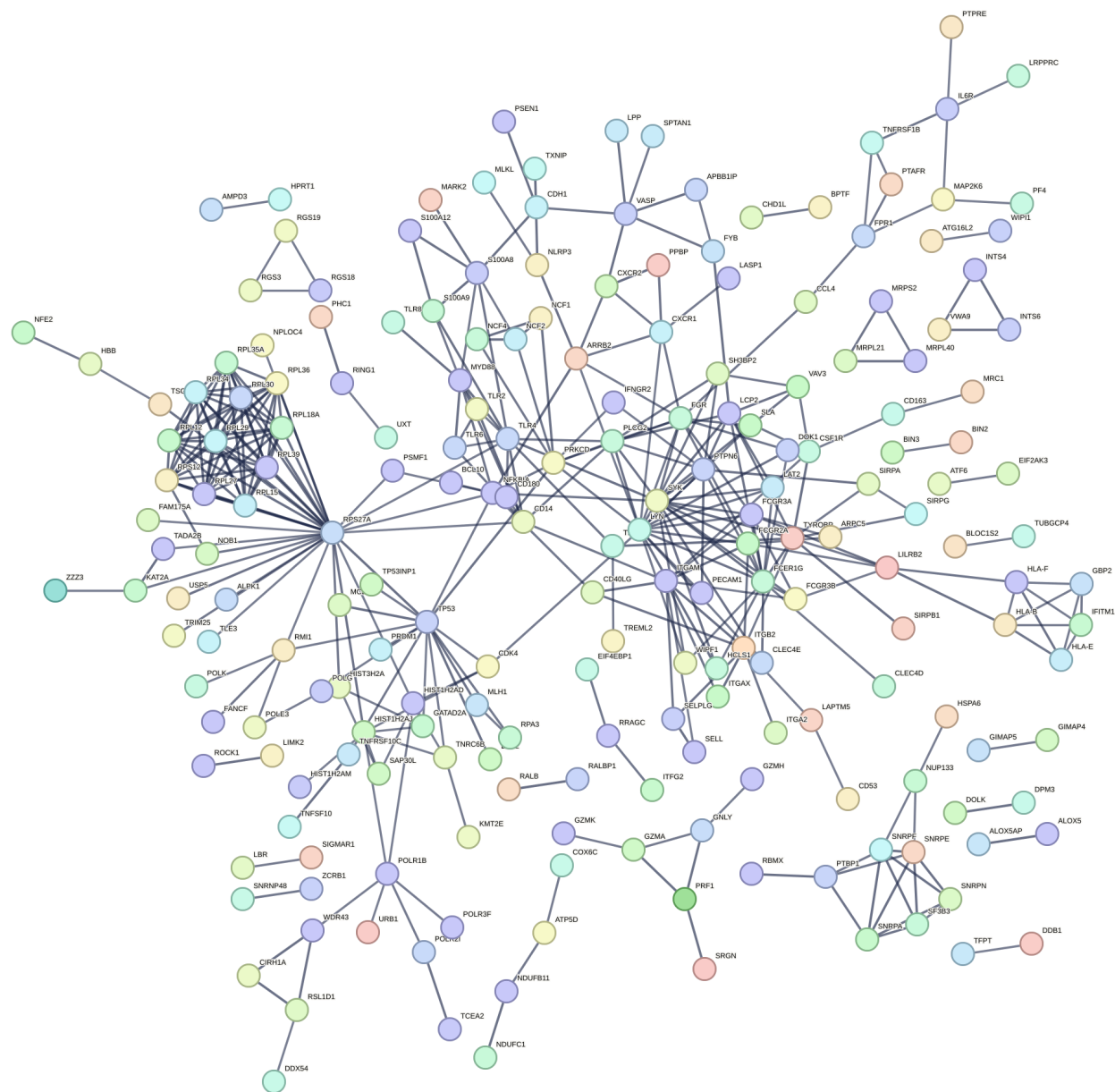

Supplement: Supplementary file 1 [file DataSheet1.zip › Suppelementary files-/Supplement Figures/S1.pdf]

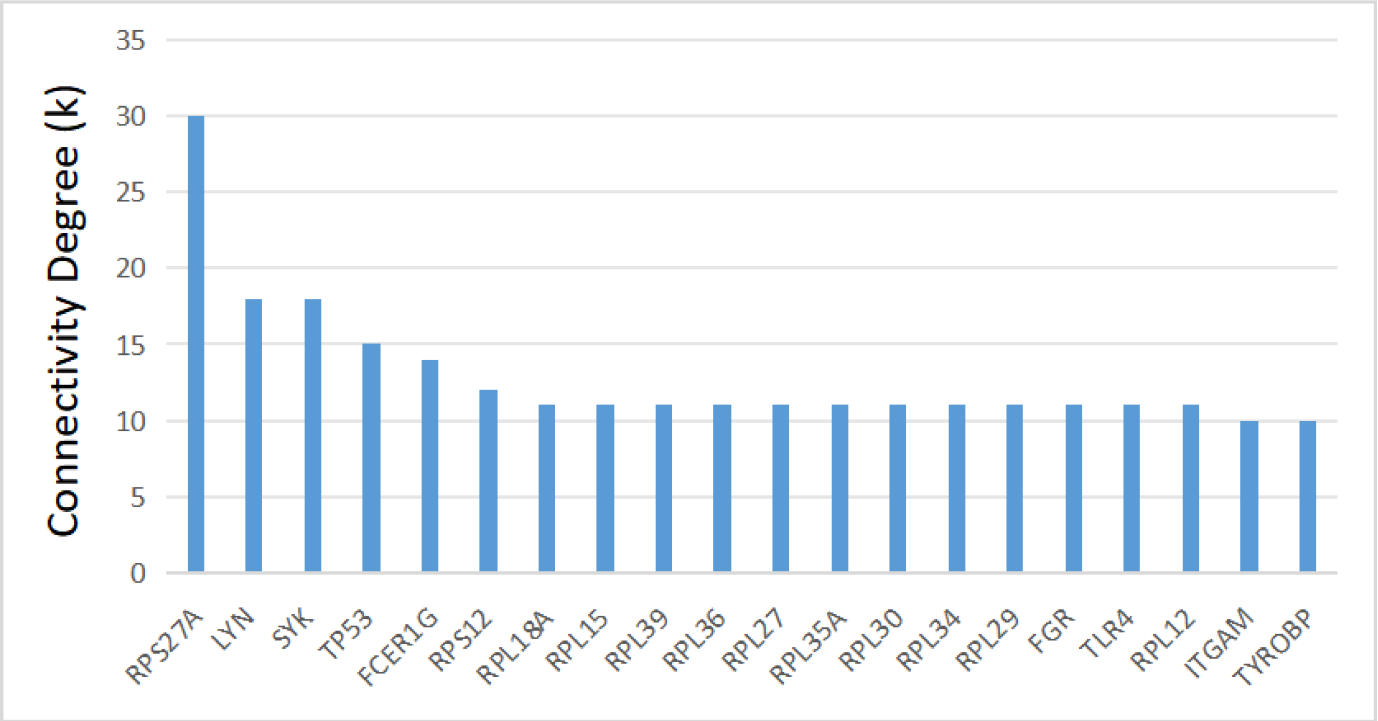

Supplement: Supplementary file 1 [file DataSheet1.zip › Suppelementary files-/Supplement Figures/S2.pdf]

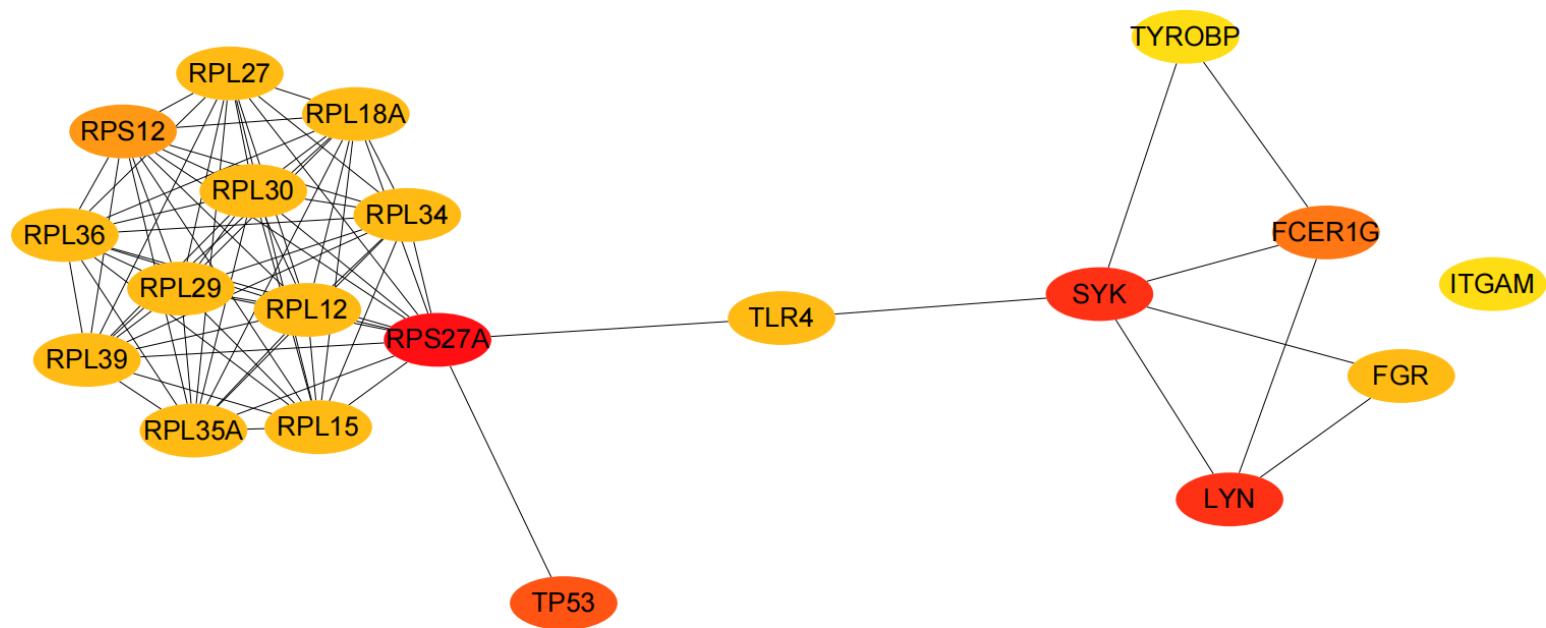

Supplement: Supplementary file 1 [file DataSheet1.zip › Suppelementary files-/Supplement Figures/S3.pdf]

**Scale independence**

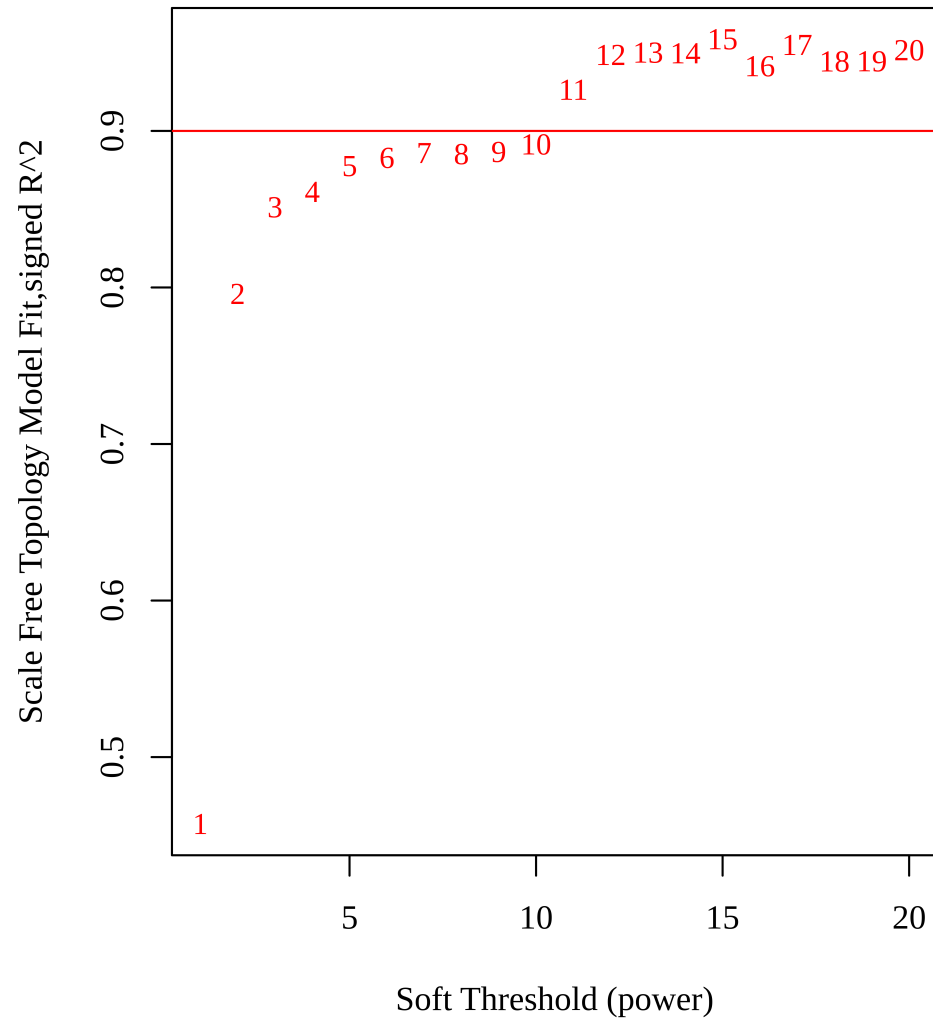

**Mean connectivity**

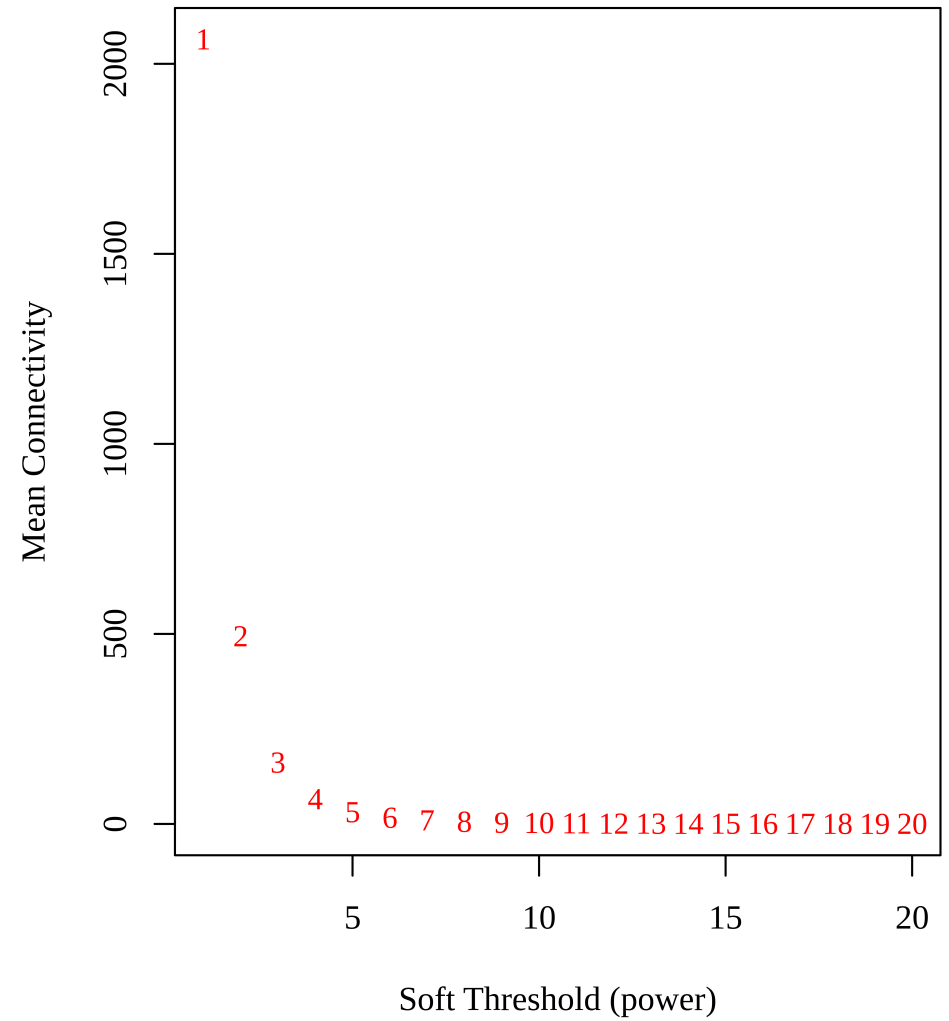

Supplement: Supplementary file 1 [file DataSheet1.zip › Suppelementary files-/Supplement Figures/S4.pdf]

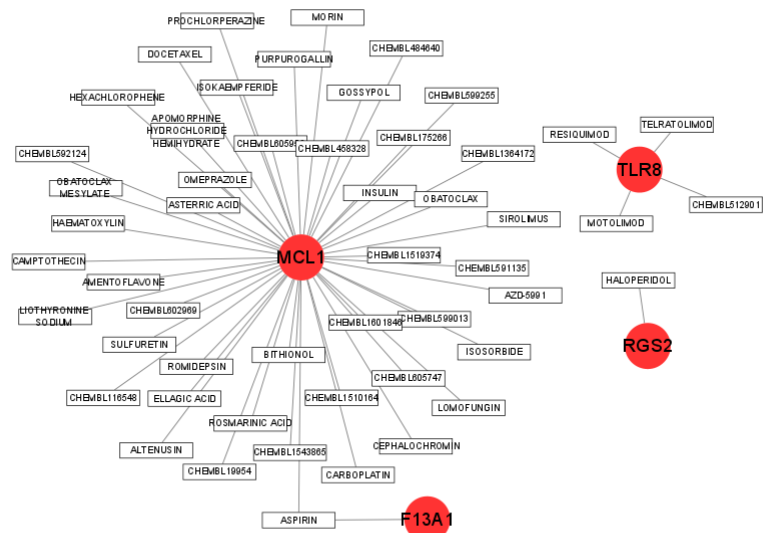

Supplement: Supplementary file 1 [file DataSheet1.zip › Suppelementary files-/Supplement Figures/S6.pdf]

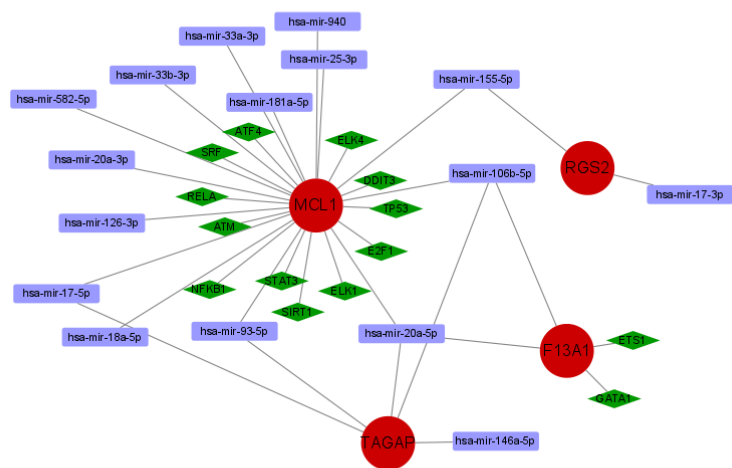

Supplement: Supplementary file 1 [file DataSheet1.zip › Suppelementary files-/Supplement Figures/S7.pdf]

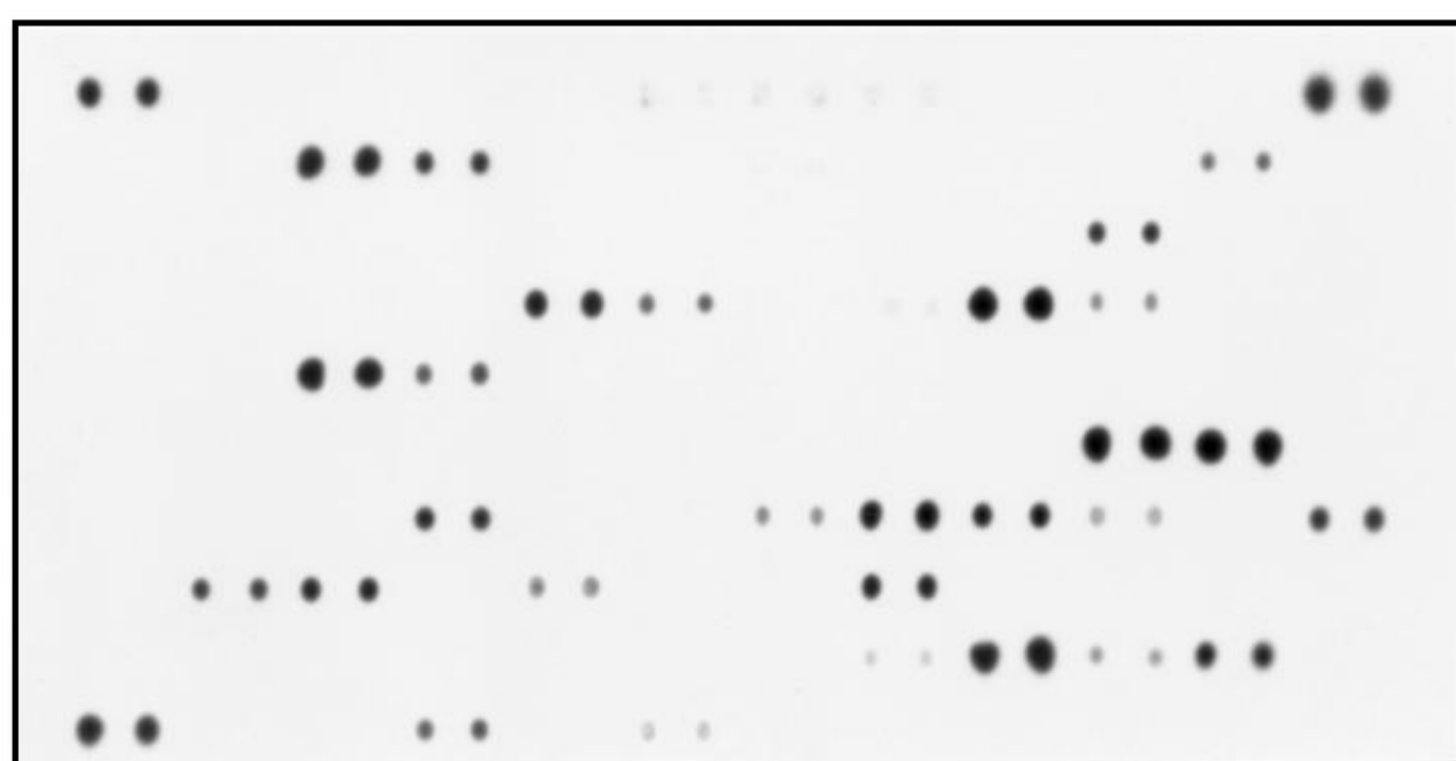

Control virus

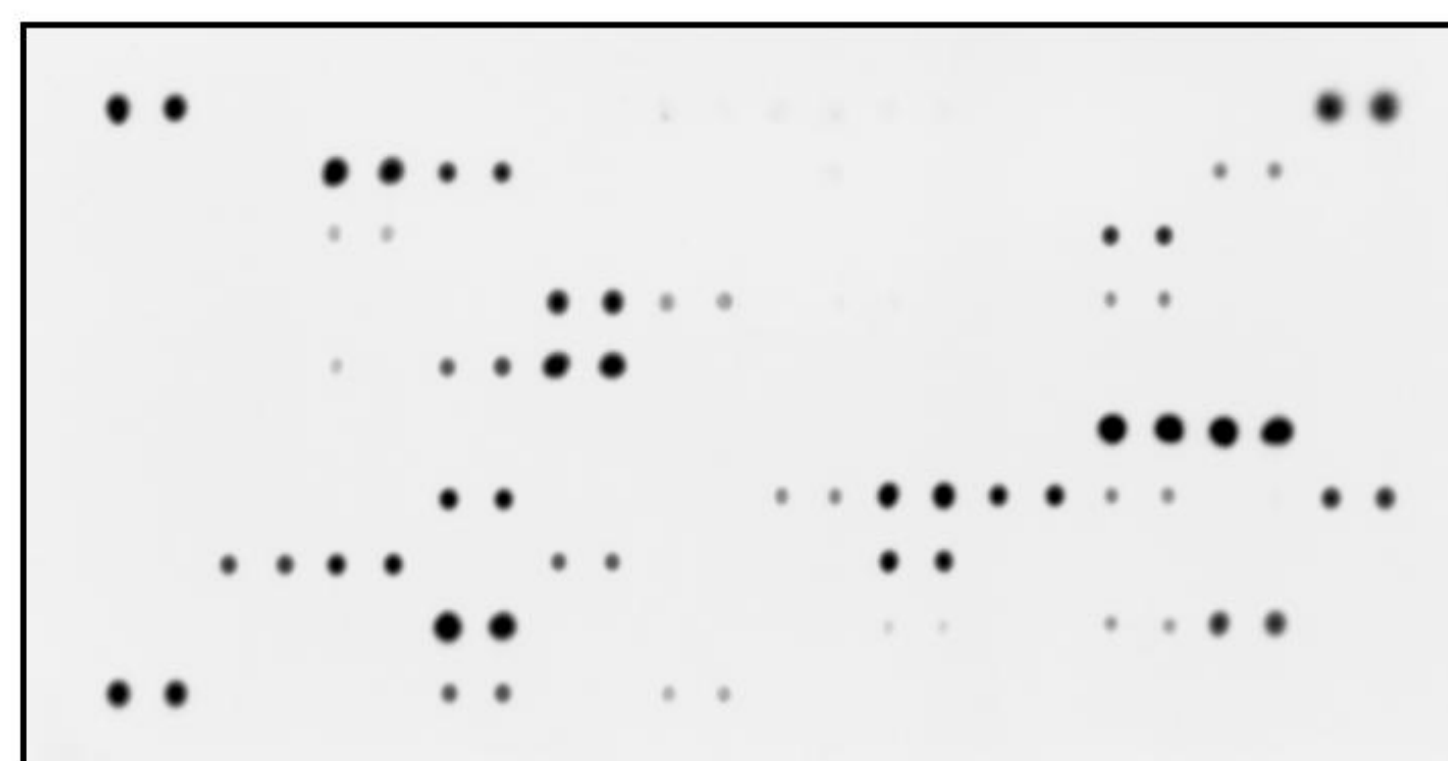

NSUN3 virus

Supplement: Supplementary file 1 [file DataSheet1.zip › Suppelementary files-/Supplement Figures/S8.pdf]
